# Conditional knockout of Na_V_1.6 in adult mice ameliorates neuropathic pain

**DOI:** 10.1038/s41598-018-22216-w

**Published:** 2018-03-01

**Authors:** Lubin Chen, Jianying Huang, Peng Zhao, Anna-Karin Persson, Fadia B. Dib-Hajj, Xiaoyang Cheng, Andrew Tan, Stephen G. Waxman, Sulayman D. Dib-Hajj

**Affiliations:** 1 Department of Neurology, Yale University School of Medicine, New Haven, CT 06510 USA; 2 Center for Neuroscience & Regeneration Research, Yale University School of Medicine, New Haven, CT 06510 USA; 3 Rehabilitation Research Center, VA Connecticut Healthcare System, West Haven, CT 06516 USA

## Abstract

Voltage-gated sodium channels Na_V_1.7, Na_V_1.8 and Na_V_1.9 have been the focus for pain studies because their mutations are associated with human pain disorders, but the role of Na_V_1.6 in pain is less understood. In this study, we selectively knocked out Na_V_1.6 in dorsal root ganglion (DRG) neurons, using Na_V_1.8-Cre directed or adeno-associated virus (AAV)-Cre mediated approaches, and examined the specific contribution of Na_V_1.6 to the tetrodotoxin-sensitive (TTX-S) current in these neurons and its role in neuropathic pain. We report here that Na_V_1.6 contributes up to 60% of the TTX-S current in large, and 34% in small DRG neurons. We also show Na_V_1.6 accumulates at nodes of Ranvier within the neuroma following spared nerve injury (SNI). Although Na_V_1.8-Cre driven Na_V_1.6 knockout does not alter acute, inflammatory or neuropathic pain behaviors, AAV-Cre mediated Na_V_1.6 knockout in adult mice partially attenuates SNI-induced mechanical allodynia. Additionally, AAV-Cre mediated Na_V_1.6 knockout, mostly in large DRG neurons, significantly attenuates excitability of these neurons after SNI and reduces Na_V_1.6 accumulation at nodes of Ranvier at the neuroma. Together, Na_V_1.6 in Na_V_1.8-positive neurons does not influence pain thresholds under normal or pathological conditions, but Na_V_1.6 in large Na_V_1.8-negative DRG neurons plays an important role in neuropathic pain.

## Introduction

Adult primary sensory neurons express five voltage-gated sodium channel isoforms (Na_V_), including TTX-sensitive (TTX-S) Na_V_1.1, Na_V_1.6 and Na_V_1.7, and TTX-resistant (TTX-R) Na_V_1.8 and Na_V_1.9 channels^1^. Na_V_1.7, Na_V_1.8 and Na_V_1.9, which are expressed preferentially in these neurons^2–4^, play critical roles in acute nociception and persistent pain in rodent models^5–9^. Identification and characterization of mutations in Na_V_1.7, Na_V_1.8 and Na_V_1.9 have confirmed causative links of these channels to human pain disorders^10–17^.

By contrast, Na_V_1.1 and Na_V_1.6, the two Na_V_ subtypes widely expressed throughout the PNS and CNS, have been linked to CNS disorders such as epilepsy^18–21^. New evidence, suggests that their roles in nociception may have been underestimated. Activation of Na_V_1.1 using a spider toxin has been shown to produce robust pain behavior and profound hypersensitivity to mechanical stimuli, establishing the first evidence linking Na_V_1.1 to acute pain and mechanical allodynia^22^. Na_V_1.6 has been implicated in oxaliplatin-induced hyper-excitability in A-fiber neurons^23^ and cold allodynia^24^. Local knockdown of Na_V_1.6 using siRNA has also been shown to reduce spontaneous neuronal activity and nociceptive behaviors in rodent models of chronic pain^25,26^. Recently, the first Na_V_1.6 gain-of-function mutation associated with a human neuropathic pain disorder, trigeminal neuralgia, was reported^27^, highlighting the need to further investigate the role of Na_V_1.6 in the pain pathway.

A challenge to the elucidation of the mechanistic basis for the role of this channel in pain in animal models is that global Na_V_1.6 null mice are juvenile lethal^28^. To examine the specific contribution of Na_V_1.6 to the excitability of DRG neurons as well as acute and neuropathic pain behaviors, we have established two conditional Na_V_1.6 knockout models with Na_V_1.8-directed or AAV-mediated Cre-Lox recombination systems, to selectively knockout Na_V_1.6 in primary sensory neurons. Our data show that Na_V_1.6 contributes a substantial fraction of the TTX-S current in both small and large DRG neurons and manifests a long half-life at nodes of Ranvier *in vivo*, and that although Na_V_1.6 has limited role in acute nociception, it contributes to the development and maintenance of neuropathic pain behavior following spared nerve injury (SNI).

## Results

### Contribution of Na_V_1.6 to TTX-sensitive current in DRG neurons

The TTX-S sodium current activates during the initial depolarization phase and is important in setting the firing threshold of action potentials in DRG neurons^29,30^. We first examined the functional consequences of Na_V_1.6 deletion in Na_v_1.8-positive neurons by comparing TTX-S current in both small (20–25 µm) and large (40–45 µm) native DRG neurons from Na_V_1.6-heterozygous control and Na_V_1.8-driven Na_V_1.6-knockout (KO) mice. The conditional Na_V_1.6 knockout mice carry the Cre recombinase which is expressed at the native Na_V_1.8 locus^31^ and Na_V_1.6 alleles flanked with LoxP sites (Na_V_1.6^flox/flox^)^32^ as well as a Cre-dependent reporter cassette (loxP-stop-loxP-tdTomato fluorescent protein). This approach allows the visual identification (red fluorescence) of functional Cre recombinase expression in Na_V_1.8-positive neurons^33^ and thus provides a marker for the deletion of the floxed Na_V_1.6 alleles.

**Figure 1:**
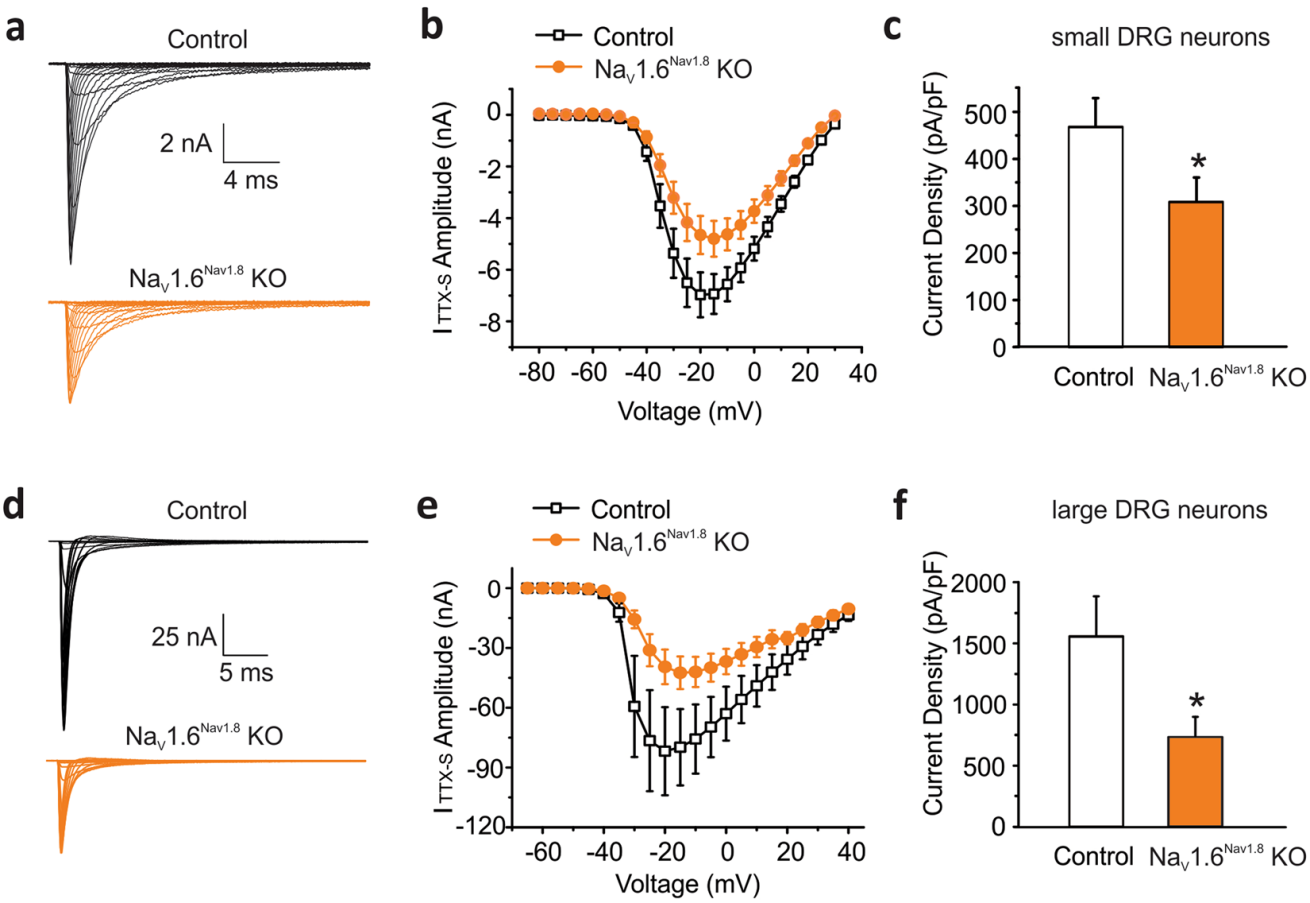
Contribution of Na_V_1.6 to TTX-sensitive sodium current in small and large DRG neurons in Na_V_1.8 driven Nav1.6 knock out mice. (**a**) Representative traces of TTX-S currents recorded in small DRG neurons from heterozygous Na_V_1.8^+/Cre^Na_V_1.6^+/flox^ (Control, black lines) and Na_V_1.8^+/Cre^Na_V_1.6^flox/flox^ (Na_V_1.6^Nav1.8^ KO, orange lines) mice. Cells were held at −100 mV and stepped to membrane potentials from −80 to +30 mV in 5 mV increments (100 ms) to record voltage-dependent sodium currents. (**b**) The current-voltage (I-V) relation curves for TTX-S sodium currents in small Control (n = 13) and Na_V_1.6^Nav1.8^ KO (n = 15) KO DRG neurons. (**c**) Current densities of TTX-S sodium currents in small Control (n = 13) and Na_V_1.6^Nav1.8^ KO (n = 15) DRG neurons. (**d**) Representative traces of TTX-S sodium currents recorded in large DRG neurons from heterozygous Na_V_1.8^+/Cre^Na_V_1.6^+/flox^ (Control) and Na_V_1.8^+/Cre^Nav1.6^flox/flox^ (Na_V_1.6^Nav1.8^ KO) mice. Cells were held at −70 mV, prepulsed to −100 mV for 500 ms, and stepped to membrane potentials from −65 mV to +40 mV in 5 mV increments (100 ms) to record the voltage-dependent sodium currents. (**e**) The current-voltage (I-V) relation curves for TTX-S sodium currents in large Control (n = 5) and Na_V_1.6^Nav1.8^ KO (n = 7) DRG neurons. (**f**) Current densities of TTX-S sodium currents in large Control (n = 5) and Na_V_1.6^Nav1.8^ KO (n = 7) DRG neurons. Data are presented as mean ± SEM. *p < 0.05 based on unpaired *t* test.

Representative traces of TTX-S currents recorded in red fluorescent small DRG neurons from Nav1.6-heterozygous control (Control: Na_V_1.6^+/flox^/Na_V_1.8^+/Cre^) and Na_V_1.8-driven Na_V_1.6 KO mice (Na_V_1.6^Nav1.8^ KO: Na_V_1.6^flox/flox^/Na_V_1.8^+/Cre^) showed a reduction of TTX-S current amplitude after Na_V_1.6 deletion (Fig. 1a). Peak TTX-S current density was calculated from the complete voltage-current relationship (Fig. 1b). Knocking out Na_V_1.6 resulted in ~34% reduction of the TTX-S current density in small DRG neurons (Control: 468 ± 59 pA/pF, n = 13; Na_V_1.6^Nav1.8^ KO 308 ± 50 pA/pF, n = 15; p = 0.0253 unpaired t test test) (Fig. 1c). By contrast, the TTX-R current density in these neurons was not affected by Na_V_1.6 deletion (Control: 59.6 ± 6.6 pA/pF, n = 12; Na_V_1.6^Nav1.8^ KO: 52.1 ± 5.3 pA/pF, n = 14).

Representative traces of TTX-S currents recorded in large red fluorescent DRG neurons from control and Na_V_1.6^Nav1.8^ KO mice showed a reduction of TTX-S current amplitude after Na_V_1.6 deletion (Fig. 1d). As shown in Fig. 1e, the average TTX-S sodium currents in these neurons were substantially reduced in Na_V_1.6^Nav1.8^ KO mice as compared to the control heterozygous littermates. The density of TTX-S sodium currents in large DRG neurons lacking Na_V_1.6 channels was significantly reduced by 53% compared to control neurons (Control: 1557 ± 328 pA/pF, n = 5; Na_V_1.6^Nav1.8^ KO: 731 ± 157 pA/pF, n = 7; *p* = 0.0317) (Fig. 1f). In contrast, the current density of TTX-R sodium currents in these large DRG neurons (mostly produced by Na_V_1.8 channels) was similar between control and Na_V_1.6^Nav1.8^ KO mice (Control: 329 ± 48 pA/pF, n = 5; Na_V_1.6^Nav1.8^ KO, 334 ± 41 pA/pF, n = 7; p = 0.931). Our results suggest that approximately 80% of the voltage-dependent sodium currents in Na_V_1.8-positive large DRG neurons are TTX-S, and more than one half of the TTX-S current is carried by Na_V_1.6 sodium channels.

Although real-time RT-PCR did not show substantial reduction in Na_V_1.6 RNA in the conditional knockout mice (Supplemental Fig. 1), the presence of red fluorescence indicates the expression of functional Cre recombinase in Na_V_1.8-positive neurons, and the reduction in the TTX-S current is consistent with the knockout of Na_V_1.6 in these neurons.

**Figure 2:**
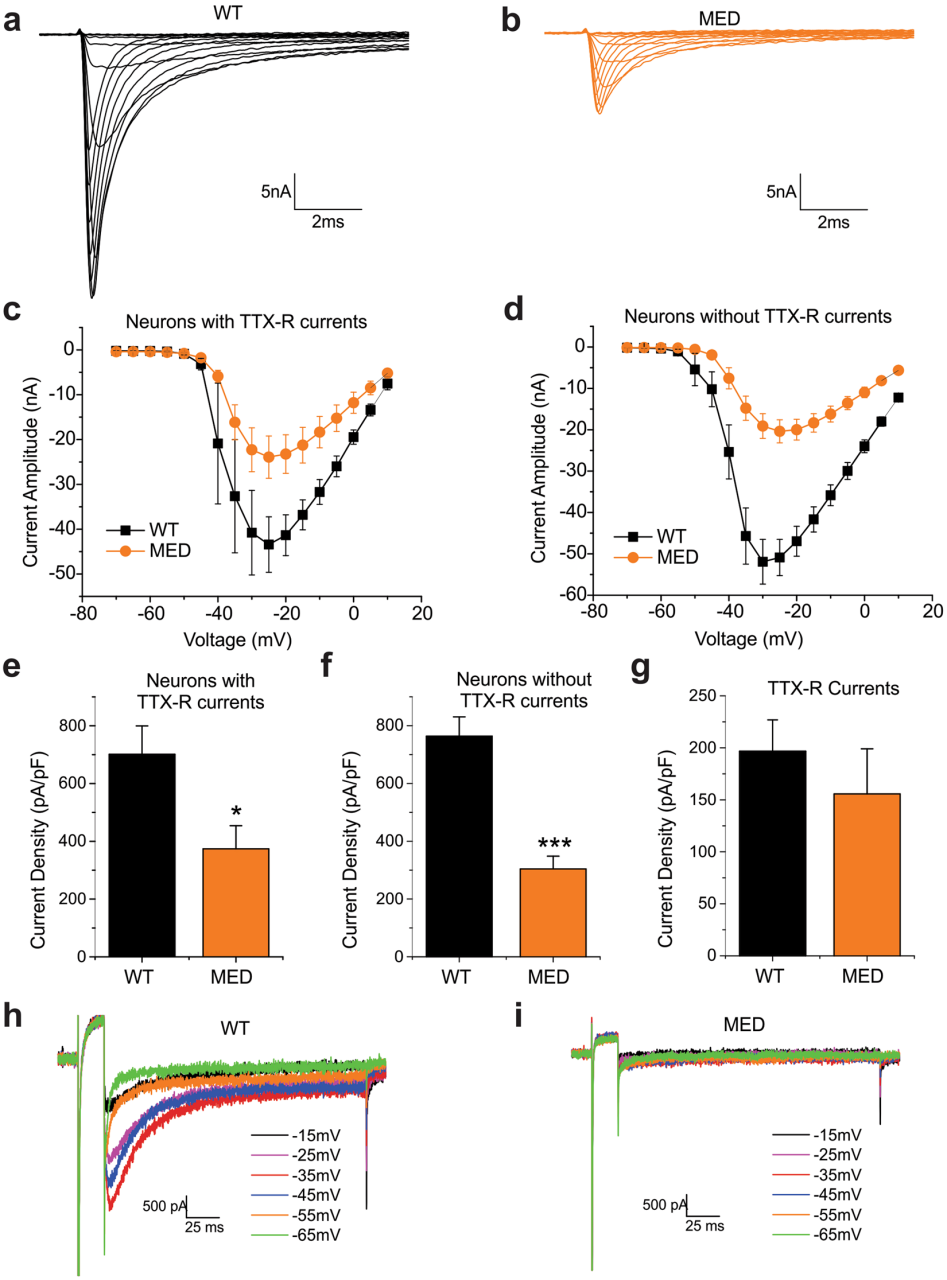
Contribution of Na_V_1.6 to TTX-sensitive sodium current in large DRG neurons in Na_V_1.6 global knockout (*SCN8a*^medtg^) mice. Representative traces of TTX-S currents recorded in large DRG neurons from (**a**) wild-type (WT) mice and (**b**) Na_V_1.6 global KO (*SCN8a*^medtg^, MED) mice. (**c**) The current-voltage (I-V) relationship curves for TTX-S sodium currents in large WT (n = 6) and MED (n = 4) DRG neurons that express TTX-R sodium currents. (**d**) The current-voltage (I-V) relationship curves for TTX-S sodium currents in large WT (n = 17) and MED (n = 16) DRG neurons that do not express TTX-R sodium currents. **(e)** Current densities of TTX-S sodium currents in large WT (n = 6) and MED (n = 4) DRG neurons that express TTX-R sodium currents. **(f)** Current densities of TTX-S sodium currents in large WT (n = 17) and MED (n = 16) DRG neurons that do not express TTX-R sodium currents. **(g)** Current densities of TTX-R sodium currents in large WT (n = 6) and MED (n = 4) DRG neurons. (**h**) Representative traces of resurgent sodium currents recorded in large DRG neurons that do not express TTX-R sodium currents from WT mice. **(i)** Representative traces show lack of resurgent sodium currents from a large DRG neurons that do not express TTX-R sodium currents from MED mice. Data are presented as mean ± SEM. *p < 0.05, ***p < 0.001 based on unpaired *t* test.

Because Na_V_1.8 channels are only expressed in a subpopulation of large DRG neurons^33^, we performed voltage-clamp recordings in a global Na_V_1.6 KO mouse strain (MED: *Scn8a*^medtg^) and homozygous wild-type littermates (WT), which were genotyped prior to tissue harvesting, in order to assess the contribution of Na_V_1.6 to the TTX-S current in neurons that do not express Na_V_1.8. The full knockout of Na_V_1.6 in *Scn8a*^medtg^ was verified at the RNA level using real-time RT-PCR (Supplemental Fig. 1) and at the protein level by the total loss of Na_V_1.6 staining at nodes of Ranvier in the sciatic nerve (Supplementa1 Fig. 2).

Representative traces of TTX-S currents recorded in large DRG neurons of WT and *Scn8a*^medtg^ mice are shown in Fig. 2a and b, respectively. We recorded TTX-R sodium currents (mostly produced by Na_V_1.8 channels under the recording conditions in these experiments) in 20% of large DRG neurons (4 out of 20 cells) from *Scn8a*^medtg^ mice and 26% (6 out of 23 cells) from wild-type littermates. In large neurons with TTX-R currents (Fig. 2c) and without TTX-R currents (Fig. 2d), the amplitude of TTX-S sodium currents was significantly reduced in *Scn8a*^medtg^ mice, compared to their WT littermates. The average TTX-S current density was reduced by 47% in *Scn8a*^medtg^ large DRG neurons that express TTX-R sodium currents (WT: 701 ± 98 pA/pF, n = 6; MED, 374 ± 80 pA/pF, n = 4; *p* = 0.045) (Fig. 2e). In large DRG neurons that do not express TTX-R sodium currents, the average TTX-S current density was reduced by 60% (WT: 764 ± 66 pA/pF, n = 17; MED: 304 ± 44 pA/pF, n = 16; *p* = 3.04E-6) (Fig. 2f). The current density of TTX-R sodium currents in large DRG neurons was unaffected in *Scn8a*^medtg^ mice (WT: 197 ± 30 pA/pF, n = 6; MED: 156 ± 43 pA/pF, n = 4; *p* = 0.443) (Fig. 2g). In addition, the current density of TTX-S sodium currents for WT cells that express TTX-R sodium currents was not significantly different from cells that do not express TTX-R currents (*p* = 0.625). Our data from *Scn8a*^medtg^ mice not only confirmed our findings in Na_V_1.8-driven Na_V_1.6 knockout mice, but also showed that Na_V_1.6 accounts for up to 60% of the TTX-S current in neurons that do not express TTX-R currents.

Nav1.6 has been shown to conduct resurgent currents in DRG neurons^34^. Representative traces for resurgent sodium currents from WT and *Scn8a*^medtg^ mice are shown in Fig. 2h and i, respectively. The average amplitude of resurgent sodium currents in WT large neurons is 1.64 ± 0.17% of the peak current. We recorded resurgent sodium currents in 82% of WT large DRG neurons that do not express TTX-R sodium currents (14 out of 17 cells) while none of the cells (0 out of 16 cells) from *Scn8a*^medtg^ mice produced resurgent currents.

### Na_V_1.8-Cre driven Na_V_1.6 KO mice manifest normal acute and persistent pain behavior

**Figure 3:**
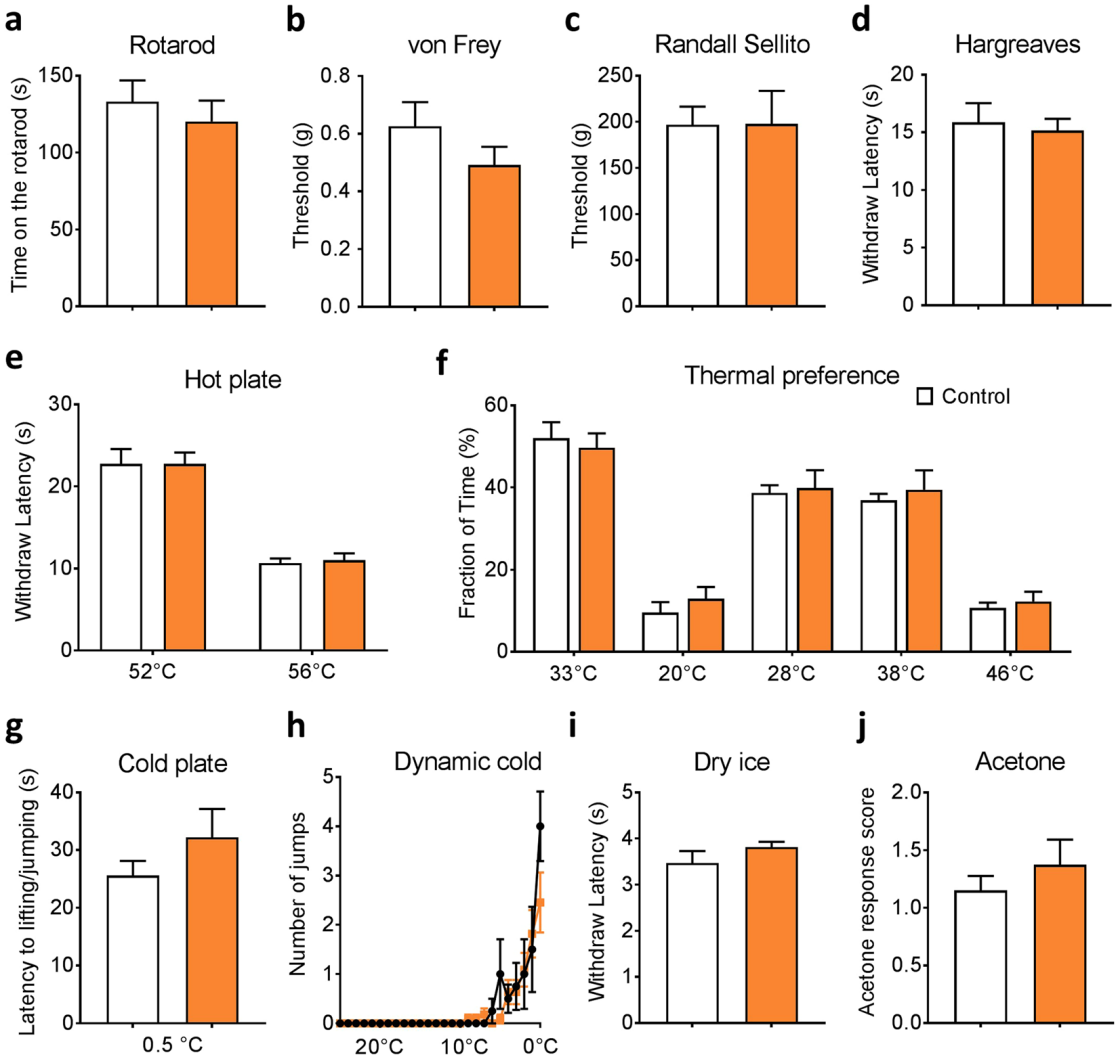
Motor and acute pain behaviors in Na_V_1.8-Cre driven Na_V_1.6 KO mice. (**a**) Motor function: rotarod test (Control, n = 6; Na_V_1.6^Nav1.8^ KO, n = 9). (**b**) Low-threshold mechanical threshold: von Frey test (Control, n = 8; Na_V_1.6^Nav1.8^ KO, n = 10). (**c**) High-threshold mechanical threshold: Randall Sellito test (Control, n = 5; Na_V_1.6^Nav1.8^ KO, n = 9). (**d**) Noxious heat (spinal reflex) threshold: Hargreaves test apparatus (Control, n = 8; Na_V_1.6^Nav1.8^ KO, n = 10). (**e**) Noxious heat thresholds (Supraspinal response) measured using hot plate test at 52 °C and 56 °C (Control, n = 12; Na_V_1.6^Nav1.8^ KO, n = 16). (**f**) Dynamic cold plate test: the number of jumps/paw lifts was measured as temperature of the plate is changed from 25 °C to 0.5 °C (Control, n = 4; Na_V_1.6^Nav1.8^ KO, n = 9). (**j**) Thermal place preference test: the percentage of time spent at a test temperature (20 °C, 28 °C, 38 °C or 46 °C) versus control temperature 33 °C (Control, n = 7; Na_V_1.6^Nav1.8^ KO, n = 9). (**g**) Noxious cold thresholds: cold plate test at 0.5 °C (Control, n = 10; Na_V_1.6^Nav1.8^ KO, n = 10). (**h**) Cooling: dry ice test (Control, n = 5; Na_V_1.6^Nav1.8^ KO, n = 9). (**i**) Cooling: acetone test (Contorl, n = 5; Na_V_1.6^Nav1.8^ KO, n = 9). Color code: Control, white; Na_V_1.6^Nav1.8^ KO, orange. Data are presented as mean ± SEM. No difference (*p* > 0.05) was observed based on unpaired *t* test.

The electrophysiological recordings in DRG neurons described above demonstrated that Na_V_1.6 deletion markedly reduced the TTX-S current in both small and large DRG neurons. Because global Na_V_1.6 is juvenile lethal, we examined whether pain behavior was altered in the Na_V_1.6^Nav1.8^ KO mice. Surprisingly, the Na_V_1.6^Nav1.8^ KO mice were phenotypically indistinguishable from their heterozygous control littermates in all behavioral tests we conducted. Conditional Na_V_1.6 knockout and control mice performed equally well in the rotarod test, suggesting that sensorimotor function was not affected (Fig. 3a). Withdrawal thresholds to mechanical stimuli assessed using von Frey and Randall Selitto tests were also not significantly different in Na_V_1.6^Nav1.8^ KO mice compared to their heterozygous control littermate (Fig. 3b,c). In addition, conditional Na_V_1.6 knockout and control mice displayed similar acute thermal pain thresholds in the Hargreaves test (radiant heat applied to hind paw) and the Hot Plate test (mice placed on a metal plate at 52 °C or 56 °C) (Fig. 3d,e). Thermal preference test was performed to examine whether deletion of Na_V_1.6 impacts avoidance behavior. Compared to control mice, no difference was observed in Na_V_1.6^Nav1.8^ KO mice at all the temperatures tested (non-noxious cool temperatures 20 °C or 28 °C, and non-noxious heat temperature 38 °C or 46 °C) (Fig. 3f).

Previous reports suggested the involvement of Na_V_1.6 in chemotherapy induced acute cooling-aggravated pain^23^. Our data showed that Na_V_1.6^Nav1.8^ KO mice and heterozygous controls had similar withdrawal latency in all acute cold pain tests, including cold plate test (0.5 °C), dynamic cold plate test (temperature dropped from 25 °C to 0.5 °C at 5 °C/min), dry ice test and acetone test (Fig. 3g–j).

**Figure 4:**
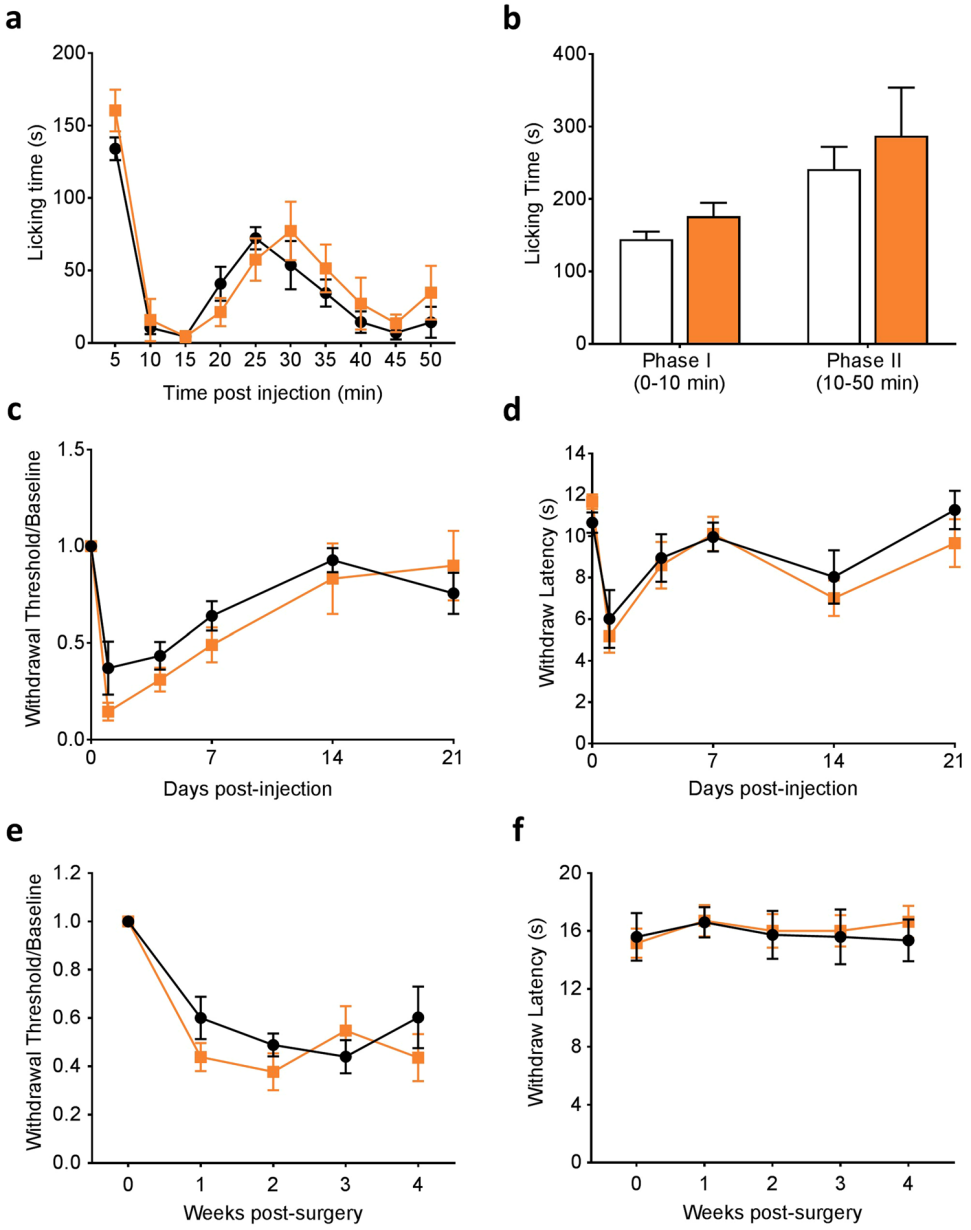
Inflammatory and neuropathic pain behaviors in Na_V_1.8-Cre driven Na_V_1.6 KO mice. (**a,b**) Behavioral responses of Nav1.8-Cre driven Nav1.6 KO mice (Na_V_1.6^Nav1.8^ KO, n = 7) and heterozygous control littermates (Control, n = 7) following intra-plantar injection of 5% formalin (10 µl). (**c**) Mechanical sensitivity measured by von Frey test following intra-plantar CFA injection (Control, n = 7; Na_V_1.6^Nav1.8^ KO, n = 9). (**d**) Thermal sensitivity measured by Hargreaves test following intra-plantar CFA injection. (Control, n = 7; Na_V_1.6^Nav1.8^ KO, n = 9). (**e**) Mechanical sensitivity measured by von Frey test following SNI (Control, n = 8; Na_V_1.6^Nav1.8^ KO, n = 10). (**f**) Thermal sensitivity measured by Hargreaves test following SNI (Control, n = 8; Na_V_1.6^Nav1.8^ KO, n = 10). Color code: Control, black; Na_V_1.6^Nav1.8^ KO, orange. Data are presented as mean ± SEM. No difference (*p* > 0.05) was observed based on unpaired *t* test.

We then examined the impact of Nav1.8 Cre-driven Na_V_1.6 knockout on inflammatory and neuropathic pain. Intra-plantar injections of formalin or complete Freund’s adjuvant (CFA) were performed to induce acute or long-term hind paw inflammation. Behavioral responses in early and late phases of formalin test in Na_V_1.6^Nav1.8^ KO mice were similar to those in control mice (Fig. 4a,b). Both control and Na_V_1.6^Nav1.8^ KO mice displayed CFA-induced mechanical hypersensitivity, which lasted for more than 7 days before the withdrawal thresholds returned to baseline values (Fig. 4c). Similarly, the Na_V_1.6^Nav1.8^ KO mice developed CFA-induced thermal hypersensitivity as their control littermates (Fig. 4d).

To study the impact of Na_V_1.6 knockout in Na_V_1.8-positive neurons on neuropathic pain behavior, we used the SNI model, in which the common peroneal and the sural branches of the sciatic nerve were transected and the tibial branch was left intact^35^. Following SNI, mechanical pain threshold was measured weekly using von Frey filaments. Na_V_1.6^Nav1.8^ KO mice developed mechanical allodynia to the same extent as their heterozygous control littermates as early as week one and maintained this lower threshold for four weeks post-surgery (Fig. 4e). As previously described using this model^35^, Na_V_1.6^Nav1.8^ KO mice did not show any abnormality in thermal thresholds (Fig. 4f).

### AAV-mediated Na_V_1.6 KO attenuates SNI-induced mechanical allodynia

The normal pain behavior of the Na_V_1.6^Nav1.8^ KO mice might be the result of several factors including compensatory effects during development and the limited knockout of Na_V_1.6 to the Na_V_1.8-positive neuronal population. To further explore the contribution of Na_V_1.6 to neuropathic pain, we used an adeno associated virus (AAV)-mediated approach to achieve adult-onset knockout of the channel that was not limited to Na_V_1.8-positive neurons DRG neurons. The advantages of using this approach include (1) the deletion of Na_V_1.6 takes place in adult mice so that any developmental compensation could be minimized; (2) AAV targets both Na_V_1.8-positive and Na_V_1.8-negative neurons; and (3) the AAV strain (AAV2/5) used in this study was previously characterized to efficiently and selectively infect primary sensory neurons when delivered intrathecally^36,37^.

AAV2/5-Cre or control AAV2/5-GFP were injected intrathecally into Na_V_1.6^flox/flox^ tdTomato mice. AAV2/5-Cre infection induces total Na_V_1.6 knockout in these neurons and produces red fluorescent protein to mark the affected neurons (Na_V_1.6^AAV^ KO), whereas neurons infected with AAV2/5-GFP will retain homozygous WT Na_V_1.6 alleles and are marked with green fluorescence (WT Control). We have confirmed in this study that intrathecal administration of AAV2/5-Cre specifically targets DRG neurons and does not infect spinal dorsal horn neurons (Supplemental Fig. 3).

**Figure 5:**
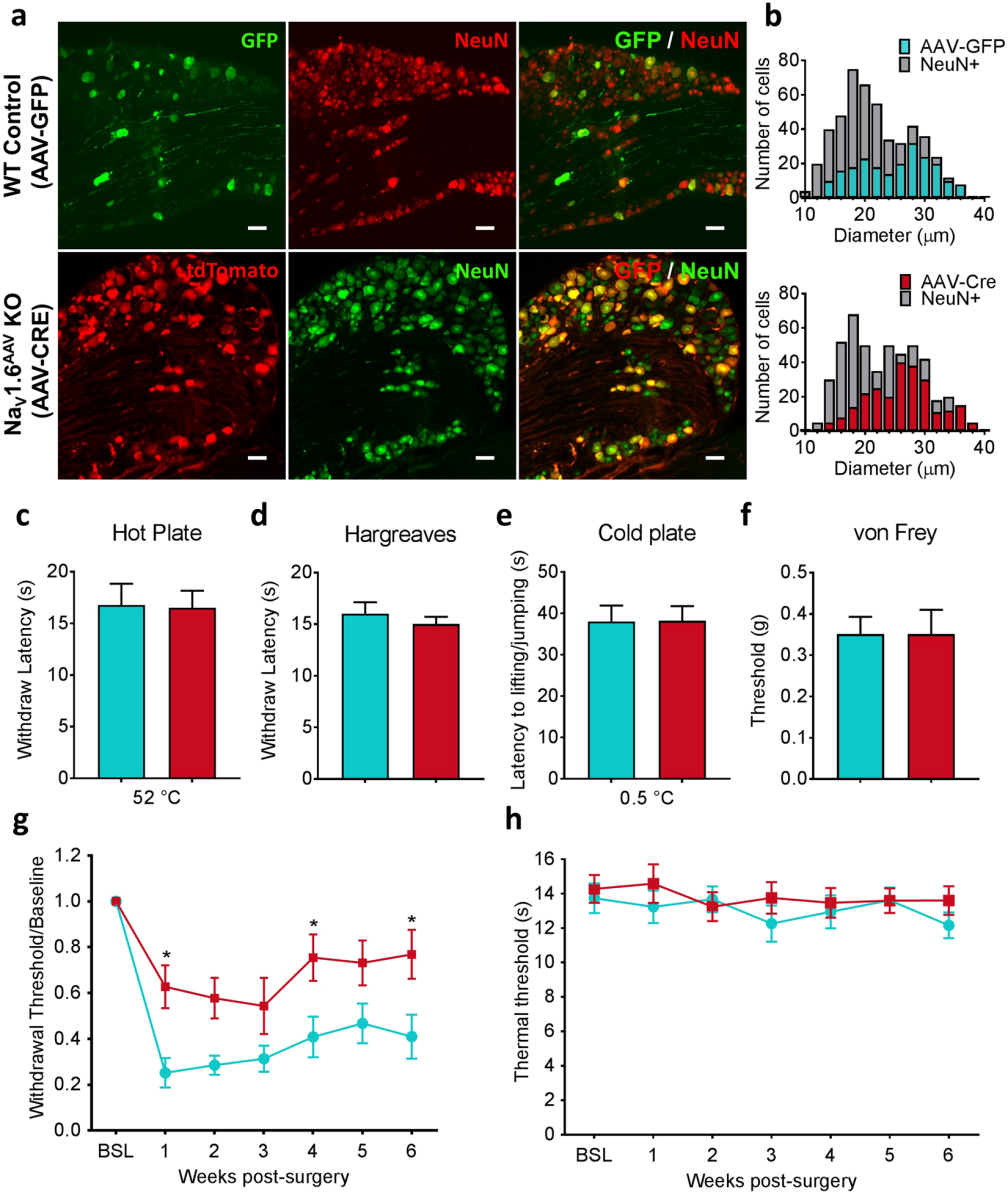
AAV-Cre mediated Na_V_1.6 knockout in adult mice attenuates SNI-induced mechanical allodynia. (**a**) Representative images of L4 DRG sections showing AAV-GFP and AAV-Cre infected DRG neurons, which are marked by GFP and tdTomato, respectively. Neurons were identified by the neuronal marker NeuN. Scale bar = 50 µm. (**b**) Size distribution of AAV-GFP and AAV-Cre infected DRG neurons. (AAV-GFP: expressed in 245 out of 485 L4 DRG neurons, n = 3; AAV-Cre: expressed in 275 out of 502 L4 DRG neurons, n = 4). (**c**) Noxious heat thresholds measured by hot plate test in AAV-GFP control (WT control, n = 8) and AAV-Cre mediated Na_V_1.6 knockout (Na_V_1.6^AAV^ KO, n = 15). (**d**) Noxious heat thresholds: Hargreaves test (WT control, n = 7; Na_V_1.6^AAV^ KO, n = 11). (**e**) Noxious cold thresholds: cold plate test (WT control, n = 5; Na_V_1.6^AAV^ KO, n = 7). (**f**) Noxious mechanical thresholds measured using von Frey test (WT control, n = 6; Na_V_1.6^AAV^ KO, n = 11). (**g**) Mechanical allodynia following SNI was measured by the von Frey test in WT control (n = 10) and Nav1.6^AAV^ KO mice (n = 12). Difference between the two groups was significant at week 1 (p = 0.0226), week 4 (p = 0.0455) and week 6 (p = 0.0336) post-surgery. (**h**) Thermal threshold measured by Hargreaves test was not changed compared to baseline in both WT control (n = 14) and Nav1.6^AAV^ KO mice (n = 15). Data are presented as mean ± SEM. *p < 0.05 based on two-way ANOVA followed by Bonferroni post hoc test.

To assess the efficiency of viral infection in DRG neurons, we used GFP/NeuN or tomato/NeuN double labelling to determine the percentage of AAV2/5 infected cells and showed that intrathecal injection of AAV2/5 produced a high infection rate (50 to 80%) in L3 to L5 DRG neurons (Fig. 5a). Unlike Na_V_1.8-driven Cre expression which was observed predominantly in small neurons and limited to a small proportion of large neurons, AAV2/5-Cre was expressed in a substantial fraction of large neurons (Fig. 5b) (AAV-GFP: expressed in 245 out of 485 L4 DRG neurons, n = 3; AAV-Cre: expressed in 275 out of 502 L4 DRG neurons, n = 4).

To determine the functional consequences of AAV-mediated knockout of Na_V_1.6 on acute pain thresholds, we assessed thermal and mechanical thresholds using hot/cold plate test, Hargreaves test and von Frey test, 3 weeks following viral vector injection, a time delay known to result in peak viral infection^36^. Compared to control animals injected with AAV2/5-GFP, Na_V_1.6^AAV^ KO mice exhibited similar behavior in all these three tests (Fig. 5c–f). We then investigated the effect of AAV-Cre mediated Na_V_1.6 knockout on neuropathic pain behavior. Intrathecal injection of AAV was conducted at the same time as SNI surgery. Thermal and mechanical thresholds were assessed after surgery once per week for six weeks using Hargreaves test and von Frey test, respectively. Compared with animals receiving control AAV2/5-GFP, Na_V_1.6^AAV^ KO mice showed a partial reduction in SNI-induced mechanical allodynia. The attenuation of mechanical allodynia was statistically significant at week 1 (p = 0.0226, two-way ANOVA followed by Bonferroni post hoc test), week 4 (p = 0.0455) and week 6 (p = 0.0336) post-surgery (Fig. 5g). As previously shown, thermal sensitivity was unaffected in this neuropathic pain model (Fig. 5h).

### AAV-mediated Na_V_1.6 knockout interrupts Na_V_1.6 accumulations at the site of injury

**Figure 6:**
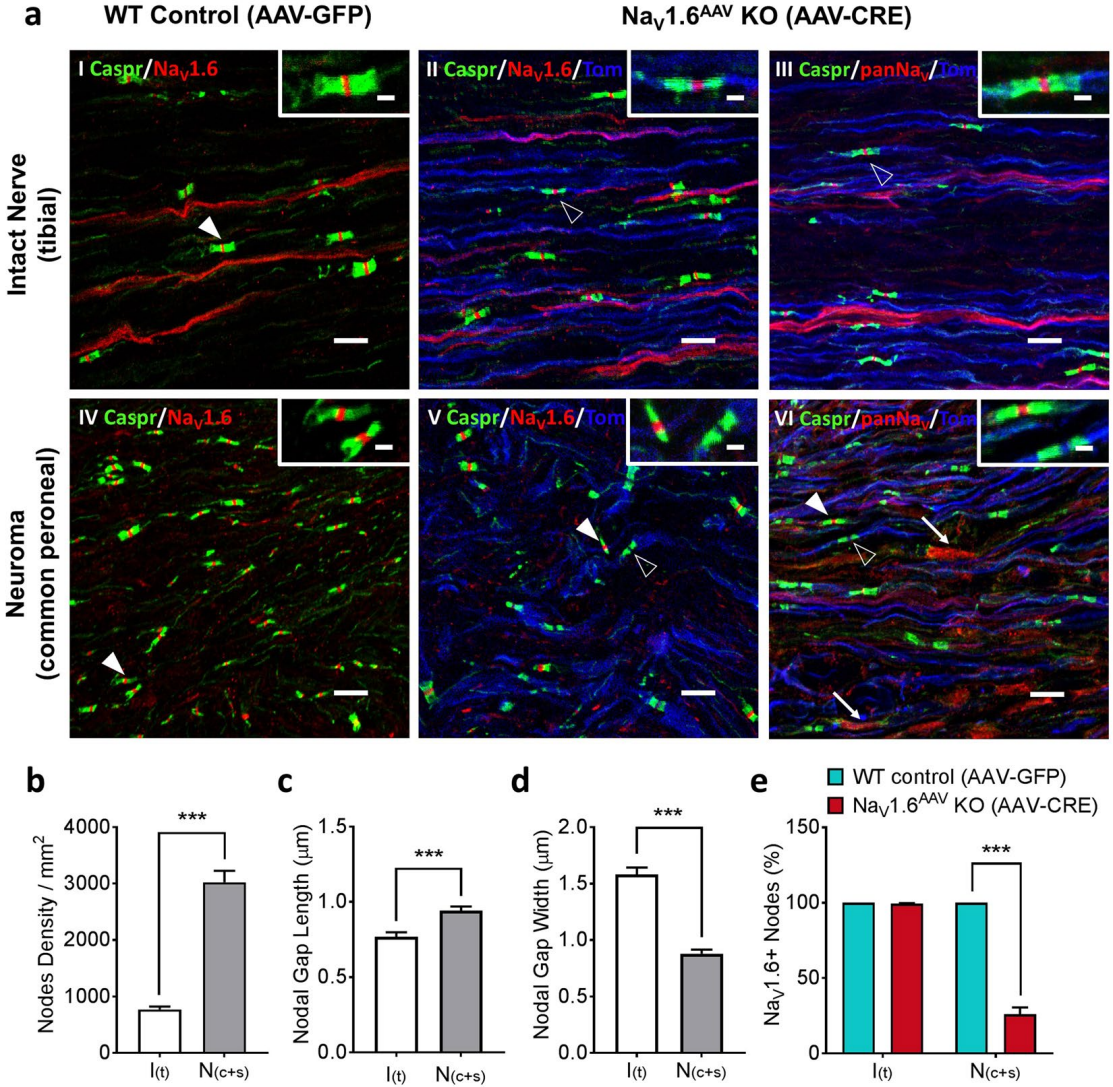
Na_V_1.6 knockout leads to empty nodes in myelinated fibers within the neuroma. (**a**) Confocal images showing Na_V_1.6 or panNa_V_ (red) and contactin-associated protein (caspr, green) immunostaining as well as tdTomato signal (blue) in intact Tibial nerve (upper row) or neuroma (Common Peroneal nerve, bottom row) from AAV-GFP infected (WT control) and AAV-Cre infected (Na_V_1.6^AAV^ KO) mice. Samples were collected 6 weeks after SNI and AAV injection. In intact nerve (**aI**) and neuroma (**aIV**) from wild-type mice, Na_V_1.6 protein is present in every node of Ranvier (filled arrowhead). In intact nerve from Na_V_1.6^AAV^ KO mice Na_V_1.6 (**aII**) protein is stable in nodes (unfilled arrow head) along tdTomato positive fibers (blue, reporter for Na_V_1.6 gene excision); similar nodal staining along tdTomato positive fibers is observed when a pan sodium channel antibody is used (**aIII**, unfilled arrow head). In neuroma from Na_V_1.6^AAV^ KO mice (**aV**), Na_V_1.6 protein is only present at nodes (filled arrow head) along tdTomato-negative fibers but absent at nodes along tdTomato-positive (blue) fibers (unfilled arrow head). Using a pan sodium channel antibody shows that the absence of Nav1.6 protein at nodes (unfilled arrow head) along tdTomato-positive (blue) fibers is not compensated by other Na_V_ isoforms (**aVI**). Filled arrow heads point to representative nodes along tdTomato-negative A-fibers and unfilled arrow heads point to representative nodes along tdTomato-positive A-fibers. Arrows point to representative non-myelinated regions within the neuroma where sodium channels accumulate following injury. Scale bar = 10 µm; Inset scale bar = 2 µm. (**b**) Comparison of average density of Na_V_1.6-positive nodes in intact nerve (white, n = 10) and neuroma (grey, n = 10). (**c**) Comparison of average nodal gap length in intact nerve (white, n = 10) and neuroma (grey, n = 10). (**d**) Comparison of average nodal gap width in intact nerve (white, n = 10) and neuroma (grey, n = 10). (**e**) Percentage of Na_V_1.6-positive neurons in neuroma after AAV-Cre (Na_V_1.6^AAV^ KO, n = 5) treatment or AAV-GFP (WT control, n = 5) treatment. Data are presented as mean ± SEM. ***p < 0.001 based on unpaired *t* test.

To investigate the molecular and cellular basis of how AAV-Cre mediated Na_V_1.6 deletion attenuates injury-induced mechanical allodynia, we studied local Na_V_1.6 distribution at the site of injury 6 weeks after SNI. In intact nerves, Na_V_1.6 staining was seen along C-fibers and at nodes of Ranvier in A-fibers (Fig. 6a.I). Following SNI, Na_V_1.6 staining was reduced in C-fibers but the presence of Na_V_1.6 was retained at all nodes in the neuroma (Fig. 6a.IV). Importantly, the density of nodes was significantly higher in the neuroma (both common peroneal and sural nerves) than in intact tibial nerve (intact: 792/mm^2^; neuroma: 3003/mm^2^; n = 10; p < 0.001, unpaired t test) (Fig. 6b). The average length and width of the nodal gap in neuroma were significantly different from those in intact nerve (length × width in intact nerve: 0.77 ± 0.03 µm × 1.58 ± 0.06 µm; neuroma: 0.94 ± 0.03 µm × 0.88 ± 0.03 µm; n = 10; p < 0.001, unpaired t test) (Fig. 6c,d). These anatomical changes may represent newly formed nodes on regenerating fibers within the neuroma.

In Na_V_1.6^AAV^ KO mice, nodal Na_V_1.6 staining was absent in tdTomato-positive fibers within neuroma (Fig. 6a.V). The percentage of nodes at neuroma which retained Na_V_1.6 was significantly reduced to 26.0 ± 2.0% in Na_V_1.6^AAV^ KO mice compared to wild-type controls (n = 5, p < 0.001, upaired t-test) (Fig. 6e). Interestingly, Na_V_1.6 signal at nodes in intact tibial nerves was not diminished for up to 6 weeks when Na_V_1.6 gene was disrupted by AAV2/5-Cre expression (Fig. 6a.II and III), suggesting that, once inserted at nodes, Na_V_1.6 proteins are highly stable, presumably because of the formation of a nodal complex with long turn-over cycle. Using a pan sodium channel antibody, we did not observe any nodal staining along tdTomato-positive fiber (Fig. 6a.VI), indicating that other sodium channel isoforms were not present at Na_V_1.6-negative nodes within neuroma. However, pan Nav staining was observed along non-myelinated axons at the neuroma (Fig. 6a.VI, arrows), which was not seen using the Na_V_1.6 specific antibody (Fig. 6a.V), suggesting the accumulation of other sodium channel isoforms in these unmyelinated fibers^38^.

### Na_V_1.6 knockout attenuates DRG neuron excitability following SNI

It has been previously established that both axotomized and intact neurons can become hyper-excitable following nerve injury^39^. We examined here whether knockout of Na_V_1.6 reduces excitability of DRG neurons. Current-clamp recordings were performed on both small and large DRG neurons cultured from Na_V_1.6^AAV^ KO (marked by red fluorescence) or control (WT, marked by green fluorescence) mice that received SNI surgery.

**Figure 7:**
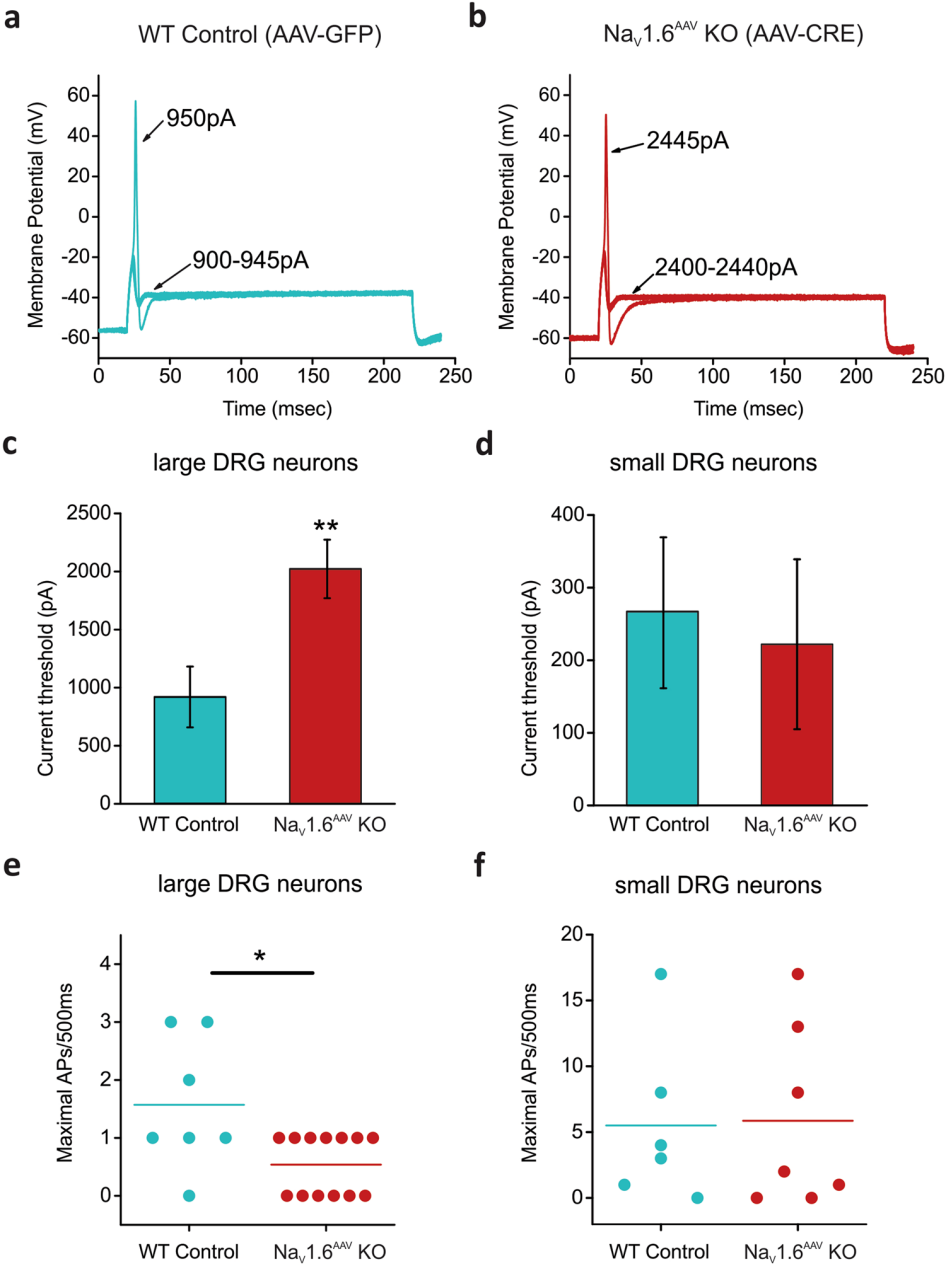
Absence of Na_V_1.6 decreases excitability of large DRG neurons following SNI. (**a**) Representative traces from a large DRG neuron expressing AAV-GFP (WT control), showing subthreshold responses to 900–945 pA current injections and subsequent all-or-none action potential by injection of 950 pA (current threshold for this neuron). (**b**) The same threshold protocol was applied to a large DRG neuron expressing AAV-CRE (Na_V_1.6^AAV^ KO). The current threshold was 2445 pA for this neuron. Arrows with numbers indicate the current amplitude used to elicit the labeled response. (**c**) Comparison of current threshold in large WT control (n = 8) and Na_V_1.6^AAV^ KO (n = 13) DRG neurons. (**d**) Comparison of current threshold in small WT control (n = 7) and Na_V_1.6^AAV^ KO (n = 7) DRG neurons. (**e**) Scatter plot of maximal action potentials in response to external current stimuli up to 2000 pA in large WT control (n = 7) and Na_V_1.6^AAV^ KO (n = 13) DRG neuron. (**f**) Scatter plot of maximal action potentials in response to external current stimuli up to 500 pA in small WT control (n = 6) and Na_V_1.6^AAV^ KO (n = 7) DRG neurons. Data are presented as mean ± SEM. *p < 0.05 based on unpaired *t* test.

Representative action potential traces from a large AAV-GFP infected WT DRG neuron (WT) in response to graded membrane potential depolarizations showed that an overshooting action potential was produced in this cell at a threshold of 950 pA (Fig. 7a). Representative action potentials recorded from a large Na_V_1.6^AAV^ KO DRG neuron are shown in Fig. 7b. An action potential did not occur in this cell until the current stimulus reached a threshold of 2445 pA. The current threshold for action potential firing differed significantly in large WT versus Na_V_1.6^AAV^ KO DRG neurons (Fig. 7c, Table 1), but were comparable in small DRG neurons (Fig. 7d, Table 2). The absence of Na_V_1.6 channels doubled the current threshold as compared to WT (WT, 918 ± 262 pA, n = 8; Na_V_1.6^AAV^ KO, 2022 ± 252 pA, n = 13; *p* = 0.00941) in large DRG neurons (Fig. 7c, Table 1). Repetitive firing was suppressed in large Na_V_1.6^AAV^ KO DRG neurons (Fig. 7e). In contrast, the maximal number of evoked action potentials in small DRG neurons was not different between control and Na_V_1.6^AAV^ KO mice (Fig. 7f). There were no significant differences (*p* > 0.05) in resting membrane potential, input resistance, or action potential amplitude for small or large DRG neurons between wild-type control and Na_V_1.6^AAV^ KO groups (Tables 1 and 2).

**Table 1:** Action potential characterization for large DRG neurons (40–45 µm) from AAV-Cre mediated Na_V_1.6 null mice and control wild-type mice.

Na_V_1.6^flox/flox^	Resting membrane potential (mV)	Input resistance (MΩ)	Action potential amplitude (mV)	Current threshold (pA)
AAV-GFP (n = 8)	−54.8 ± 2.5	66.3 ± 9.8	108 ± 3.9	918 ± 262
AAV-CRE (n = 13)	−58.7 ± 0.73	55.7 ± 7.1	116 ± 2.6	2022 ± 252**
*p*-value	0.0913	0.381	0.0930	0.00941

**Table 2:** Action potential characterization for small DRG neurons (20–25 µm) from AAV-Cre mediated Na_V_1.6 null mice and control wild-type mice.

Na_V_1.6^flox/flox^	Resting membrane potential (mV)	Input resistance (MΩ)	Action potential amplitude (mV)	Current threshold (pA)
AAV-GFP (n = 7)	−55.4 ± 2.3	140 ± 27	111 ± 5.2	267 ± 104
AAV-CRE (n = 7)	−50.5 ± 2.7	539 ± 199	110 ± 4.8	222 ± 117
*p*-value	0.192	0.0707	0.850	0.779

## Discussion

Dynamic changes in Na_V_ channel expression and function contribute to neuronal hyper-excitability following peripheral nerve injury^40,41^. Although Na_V_1.6 has recently been implicated in pain in humans^27^, and in animal models^23–26^, the specific contribution of Na_V_1.6 to the TTX-S current in DRG neurons and to neuropathic pain in knockout models has thus far not been reported. We report in this study that Na_V_1.6 contributes 34% of the TTX-S sodium current in small DRG neurons, 53% of this current in large Na_V_1.8-positive DRG neurons and 60% of this current in Na_V_1.8-negative DRG neurons. We report that Na_V_1.8-Cre driven knockout of Na_V_1.6 does not alter acute, inflammatory or neuropathic pain behaviors, while the adult-onset knockout of Na_V_1.6 via AAV-Cre ameliorates mechanical allodynia in the SNI model but without altering acute pain thresholds. We also show that SNI leads to an increase in the density of nodes of Ranvier in myelinated axons within the neuroma, and that Na_V_1.6 channels are stable at nodes for several weeks after the gene is knocked out. In parallel with the recovery of pain thresholds in the AAV-Cre mediated Na_V_1.6 knockout mice following SNI, excitability of large DRG neurons, but not small DRG neurons, was attenuated. Our data support an important role of Na_V_1.6 in large DRG neurons in neuropathic pain.

We have used three independent criteria to conclude that the expression of Cre recombinase induces the deletion of Na_V_1.6 floxed alleles in DRG neurons: (a) production of red fluorescence by a Cre-dependent tdtomato reporter cassette; (b) reduction of the TTX-S current and no change in the TTX-R current in neurons in which Na_V_1.6 is expected to be knocked out (red fluorescent neurons); (c) demonstration of the loss of nodal staining in myelinated fibers in which Na_V_1.6 is expected to be knocked out (red fluorescent fibers). The specificity of the Na_V_1.6 antibody was unequivocally validated in the mouse with global Na_V_1.6 knockout (Scn8a^medtg^, Supplemental Fig. 1). Taken together, this data supports our conclusion that the floxed Na_V_1.6 alleles are deleted in the DRG neurons that express Cre recombinase either from the Na_V_1.8 locus or via the AAV-Cre administration.

Na_V_1.6 is an abundant sodium channel in medium-large DRG neurons which produce myelinated A-fibers, and is also expressed in small DRG neurons that produce unmyelinated C-fibers^42–44^. We show here that deleting Na_V_1.6 in Na_V_1.8-positive neurons which represents 92% of small DRG neurons^33^ reduces the TTX-S current in these neurons by 34%. On the other hand, Na_V_1.6 channels contribute 47% of the TTX-S current in large Na_V_1.8-positive neurons and 60% in large Na_V_1.8-negative neurons. This functional data is consistent with molecular data using *in situ* hybridization which shows higher expression levels of Na_V_1.6 in medium and large DRG neurons over small DRG neurons^43,45^. Our functional data are also consistent with single-cell RNAseq profiling of mouse DRG neurons showing relatively higher Na_V_1.6 RNA in low threshold mechanoreceptors (LTMR) and proprioceptors, peptidergic nociceptor type 2 and C-LTMR, and very low levels in nonpeptidergic nociceptors^46^. The contribution of Na_V_1.6 to 34% of the TTX-S current in small Na_V_1.8-positive DRG neurons is consistent with the loss of 70% of the TTX-S current in small DRG neurons from Na_V_1.8-Cre driven knockout of Na_V_1.7^47^. Thus, Na_V_1.6 and Na_V_1.7 appear to account for all the TTX-S current in small DRG neurons. In large DRG neurons, Na_V_1.6 channels contribute the majority of the TTX-S current, making up 53% of the TTX-S current in large Na_V_1.8-positive neurons and 60% in large Na_V_1.8-negative neurons. The balance of the TTX-S current in large DRG neurons after knocking out Na_V_1.6 is likely mediated by Na_V_1.1 and Na_V_1.7^1^. The substantial contribution of Na_V_1.6 to TTX-S current in both small and large DRG neurons suggests a role for this channel in the transmission of pain signals.

The Na_V_1.8-Cre-driven Na_V_1.6 knockout mice were phenotypically indistinguishable from their littermate controls in acute, inflammatory and neuropathic pain models. The unaltered pain phenotype is unexpected given the reported role of Na_V_1.8-positive neurons in acute and inflammatory pain^5,6,9,48^, and the contribution of Na_V_1.6 to the amplitude of the compound action potential in the sciatic nerve^44^. Our observation suggests that in the absence of Na_V_1.6 in Na_V_1.8-positive neurons, other Na_V_ isoforms are sufficient to mediate neuronal responses to noxious stimuli.

There are several alternative explanations for the unaltered pain thresholds when Na_V_1.6 deletion is limited to Na_V_1.8-positive neurons. Compensatory dysregulation of other molecules may have occurred during development, for example, the TTX-S current was up-regulated in Na_V_1.8-null mutant mice^6^, and Na_V_1.7 knockout was reported to increase met-enkephalin expression^49^. Additionally, Na_V_1.8 expression has been reported in CNS nuclei of naïve mice^50^, thus Na_V_1.8-Cre driven Na_V_1.6 knockout in these neurons may confound the effects of Na_V_1.6 deletion in primary afferents. Moreover, normal neuropathic pain has also been reported to be retained following Na_V_1.8 knockout, Na_V_1.7/Na_V_1.8 double knockout or genetic ablation of Na_V_1.8-positive neurons^5,6,51^. It is possible that Na_V_1.8-positive neurons, most of which are small DRG neurons, might have limited effect on neuropathic pain behavior.

AAV-mediated Na_V_1.6 knockout has adult onset and is not limited to Na_V_1.8-positive neurons, thus providing an opportunity to avoid the confounds of the Na_V_1.8-Cre approach to investigate the role of Na_V_1.6 in pain. Injured myelinated Aβ afferents generate most of the ectopic discharges around the time of pain onset^52,53^, and sodium current block in large myelinated fibers has been shown to suppress mechanical allodynia in a few neuropathic pain models^54^. We report here that AAV-mediated Na_V_1.6 knockout, likely in large neurons, significantly attenuates SNI-induced mechanical allodynia. The partial attenuation of pain hypersensitivity observed in this study should be considered an underestimation of the contribution of Na_V_1.6 to mechanical allodynia due to the incomplete knockout of Na_V_1.6 using this approach (range of infection rate: 50–80% of DRG neurons).

Traumatic injury to peripheral nerves can lead to the formation of painful neuromas, tangled masses of blind-ending axons and proliferating Schwann cells with connective tissue which can produce spontaneous pain, brush-evoked allodynia and pinprick hyperalgesia^55^. Ectopic firing arising from myelinated Aβ fibers within neuromas has been implicated in neuropathic pain^56^. The contribution of TTX-S channels, including Na_V_1.6, to ectopic firing at neuromas is supported by the findings that treatment with TTX at concentrations not likely to affect TTX-R channels reduces this ectopic activity^57^. We have shown here that Na_V_1.6 accumulates at nodes of Ranvier that form *de novo* in neuromas. A similar accumulation of Na_V_1.6 has been observed after infraorbital nerve lesion in a rat trigeminal pain model^58^. Although we did not determine the reduction in RNA levels of Na_V_1.6 in AAV-Cre treated mice, the functional loss of Na_V_1.6 at *de novo* nodes along tdTomato-positive fibers in the neuroma and increased threshold for action potential firing and reduced evoked firing in large DRG neurons following SNI injury is consistent with the knockout of the floxed Na_V_1.6 alleles in these mice. Our data also suggest that Na_V_1.6 is the only isoform expressed at the newly formed nodes within neuromas, because we did not detect a signal at these nodes using a pan-sodium channel antibody. Na_V_1.6 immunostaining remained at nodes in intact Tibial fibers for up to 6 weeks following Na_V_1.6 deletion, the longest time tested in this study, reflecting the stability of Na_V_1.6 channels once inserted at nodes. Taken together, these data suggest that new nodal formation at neuroma and Na_V_1.6 accumulation at these nodes likely contribute to the development of injury-induced neuropathic pain.

**Figure 8:**
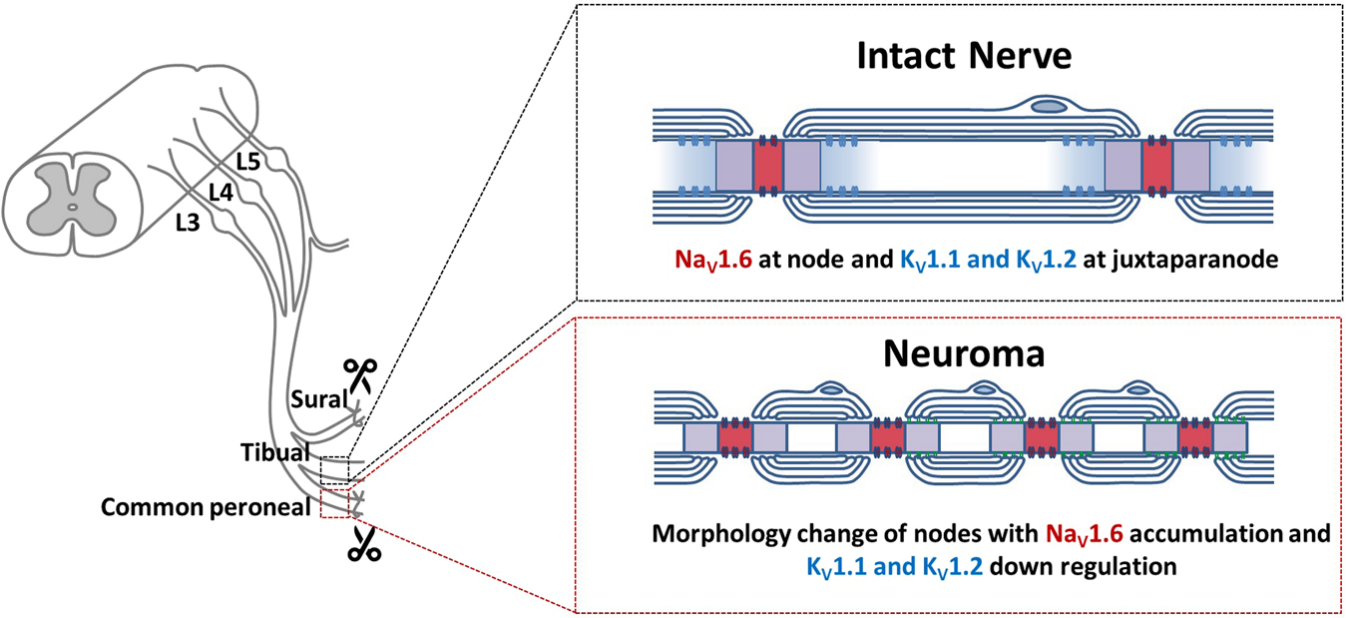
Schematic illustration of myelinated axons in intact nerve and regenerating myelinated axons within the neuroma. In intact sciatic nerve (the Tibial nerve in the SNI model), Na_V_1.6 is clustered at nodes of Ranvier with K_V_1.1 and K_V_1.2 present at juxta-paranodal regions. Regenerating myelinated axons within the neuroma show higher density of nodes of Ranvier with Na_V_1.6 as the only Na_V_ isoform present at these nodes, and are characterized by elongated nodal length and smaller fiber caliber and loss of K_V_1.1 or K_V_1.2 at juxta-paranodal regions. The increased nodal density with the accumulation of Na_V_1.6 at these nodes and the loss of K_V_1.1 and K_V_1.2 at the juxta-paranodes, and the shorter intermodal distance increase the probability of secure conduction of action potentials that are initiated in the injured fibers and the probability of burst and spontaneous firing, contributing to hyperexcitability of DRG neurons and mechanical allodynia.

Injury causes an increase in the excitatory drive which is manifested as hyperexcitability of DRG neurons that underlie pain. We show in this study an increased density of nodes of Ranvier at the neuroma with a robust nodal Na_V_1.6 signal, comparable to that observed at nodes of uninjured fibers, and short intermodal distance. We also observed the absence of K_V_1.1/K_V_1.2 at juxta-paranodes (Supplemental Fig. 4). The significant reduction of K_V_1.1/K_V_1.2 in myelinated fibers in the mouse SNI model is consistent with a recent report of dynamic redistribution of K_V_1 channels within nodes following injury^59^. The increased nodal density with normal Na_V_1.6 levels, and the loss of K_V_1.1/K_V_1.2 at the juxta-paranodes are predicted to increase the safety factor for reliability and temporal fidelity of action potential conduction at the neuroma^60^. These changes enhance the excitatory drive which is predicted to contribute to mechanical allodynia in the SNI model (Fig. 8).

We have previously shown that Na_V_1.3, Na_V_1.7 and Na_V_1.8 channels accumulate within blind-ends of injured axons in painful human neuromas and in a rat model and suggested that they contribute to ectopic at the neuroma^38,61^. We provide here clear evidence for markedly increased density of nodes of Ranvier, reducing the intermodal distance, with dense nodal staining for Na_V_1.6 at the site of nerve injury. Reduced intermodal distances are known to increase conduction safety factor and can contribute to repetitive firing^62^. We suggest that the ectopic action potential initiation that may arise at the nerve endings supported by the accumulation of sodium channels, has a higher probability of propagating along the injured myelinated fibers because of the closed spacing and accumulation of Na_V_1.6 at nodes within the neuroma, and thus contribute to afferent hyperexcitability in the injured nerve.

In conclusion, we report here that Na_V_1.6 contributes substantially to the TTX-S current in large and small DRG neurons. Na_V_1.6 knockout in Na_V_1.8-positive neurons does not alter acute, inflammatory or neuropathic pain behaviors. By contrast, AAV-mediated Na_V_1.6 knockout in adult mice partially attenuates SNI-induced mechanical allodynia as early as one week following injury. In parallel with ameliorated mechanical pain, we show that AAV-Cre mediated Na_V_1.6 knockout prevents Na_V_1.6 accumulation at *de novo* formed nodes of Ranvier adjacent to the site of nerve injury, and reduces excitability of large DRG neurons. Taken together, these data provide strong evidence for a role of Na_V_1.6 in large neurons in neuropathic pain.

## Materials and Methods

### Animals and surgical procedures

All animal experiments were conducted in accordance with the NIH Guide for the Care and Use of Laboratory Animals and were approved by the IACUC of the Veterans Administration Connecticut Healthcare System. Adult mice (2 to 4 months) of both sexes were used in this study. Mouse strains with floxed *Scn8a* (Na_V_1.6^flox/flox^)^32^ and *Scn8a*^medtg^ (global Na_V_1.6 KO)^28^ were a generous gift from Dr. Miriam Meisler (University of Michigan). Na_V_1.6 global knockout mice survive up to two weeks, and the homozygous KO mice become visually identifiable around P12 (smaller size and hindlimb paralysis), whereas wildtype and heterozygous mice are phenotypically indistinguishable. Scn8a^medtg^ mice and their wildtype littermates were genotyped before harvesting their tissues. Na_V_1.8-Cre driven Na_V_1.6 knockout (Na_V_1.8^+/Cre^, Na_V_1.6^flox/flox^) and heterozygous (Na_V_1.8^+/Cre^, Na_V_1.6^flox/+^) mice (21 to 33 g) were used in one set of experiments. In the other set of experiments, homozygous Na_V_1.6-floxed (Na_V_1.6^flox/flox^) mice (21 to 33 g) were separated into two groups which received intrathecal injection of AAV viruses that carry the Cre recombinase (AAV-Cre) or GFP (AAV-GFP) to generate AAV-Cre mediated Na_V_1.6 knockout and wild-type control mice. All mice carried a Cre-reporter cassette (loxP-stop-loxP-tdTomato fluorescent protein), which leads to the production of a red fluorescent protein in neurons that express a functional Cre recombinase^33^.

#### Intrathecal injection

Under isoflurane (1–3% in oxygen) anesthesia, a laminectomy was performed between lumbar levels L5 and L6 to expose the thecal sac. This allowed visual confirmation that the needle entered the intrathecal space without damaging the spinal cord. To avoid injury to the underlying neural tissue, the needle was kept at midline and slowly inserted at a shallow angle (less than 30°) underneath the dura. Virus particles in a 5 μl volume were injected using a 10 μl Hamilton syringe with a 33-gauge needle. After injection, the paraspinal muscle and skin layers were closed using 6–0 monofilament nylon sutures (ETHICON). The viruses used in this study were as follows: AAV2/5-CMV-Cre, AAV2/5-CMV-eGFP (Gene Transfer Vector Core, IA). Virus titers were on the order of 1 × 10^13^ viral genomes (vg)/ml.

#### SNI

Procedures were performed as previously described^35^ immediately after the intrathecal injection of AAV. Briefly, an incision (1 cm) was made on the disinfected surgical field (left lateral mid-thigh) and the underlying muscles were separated to expose the three branches of the sciatic nerve. The common peroneal and sural branches were tightly ligated with 6-0 silk sutures (DemeTECH). The ligated branches were then transected and approximately 2 mm of the distal nerve stump was excised to minimize regeneration. Efforts were made to minimize any contact with the spared tibial branch. In sham animals, the sciatic nerve and its branches were exposed identically but were neither ligated nor transected. The muscle and skin layers were then closed with 6-0 monofilament nylon sutures (ETHICON).

### Behavioral assays

All animals were acclimatized to the testing apparatus in three one-hour habituation sessions. One experimenter blind to genotypes or treatment groups performed all behavioral tests.

#### Rotarod test

To assess sensorimotor coordination, animals were placed onto the Rotarod apparatus running at 4 r.p.m., which was then accelerated to a maximum of 40 r.p.m. in 150 s. The time spent on the rotating rod was recorded with the cutoff time of 180 s.

#### von Frey test

To measure mechanical thresholds, mice were placed on an elevated wire grid and the plantar surface of the left paw (ipsilateral to the surgery/injection) of each animal was presented with a series of calibrated von Frey hairs (Stoeling, Wood Dale, IL). The 50% withdrawal threshold was determined using the “up-down” method^63^.

#### Randall-Sellito test

Noxious mechanical thresholds were measured using the Randall-Sellito electronic algesimeter (IITC 2500 Digital Paw Pressure Meter, IITC Life Science, Woodland Hills, CA). Each animal received 5 min of handling before being immobilized in the holding chamber. A uniformly increasing pressure was applied to the tail via a blunt conical probe until a withdrawal response resulted. The mechanical stimulation was repeated 3 times with 15 min separation between consecutive tests. The cut-off value was set at 500 g to prevent tissue damage.

#### Hargreaves test

To assess noxious heat threshold, the plantar paw surface was exposed to radiant heat using a Hargreaves apparatus (IITC, Woodland Hills, CA) and the paw withdrawal latency was measured. The heat stimulation was repeated 5 times with 15 min separation between consecutive tests. The cut-off value was set at 30 seconds to prevent tissue damage.

#### Hot/Cold plate

To assess thermal sensitivity, animals were placed on a metal plate with a constant temperature of 52 °C, 56 °C or 0.5 °C. The animal was removed from the plate immediately upon licking or flicking a hindpaw or if no response occurred within a predetermined cut-off time (30 s for hot plate and 120 s for cold plate).

#### Dynamic cold plate

To assess the noxious cold tolerance, animals were placed on the test arena with the surface temperature progressively cooled from 25 °C to 0.5 °C at a rate of −5 °C/min and their nocifensive behaviors (jumping and paw lifting) were noted.

#### Acetone test

50 µl of acetone was sprayed onto the plantar surface of the hindpaw using a blunt rubber tube attached to a 1-ml syringe. Behavioral responses were recorded over 30 s for each of five acetone applications. The responses were graded to a three-point scale: 0 = a brisk lifting, licking, or flicking of the hindpaw, which subsides immediately; 1 = lifting, licking, and/or flicking of the hindpaw, which continues beyond the initial application, but subsides within 5 s; 2 = protracted, repeated lifting, licking, and/or flicking of the hindpaw^64^.

#### Dry ice test

A dry ice pellet was made by compressing fine dry ice powder into a 3-ml syringe (with top cut off) and was then firmly pressed against the glass surface underneath the mouse hind paw until a withdrawal response resulted^65^. The testing procedure was repeated 3 times with 15 min separation between consecutive tests.

#### Two-plate thermal preference test

The thermal preference apparatus consisted of two cold/hot plates (Bioseb, Chaville, France) placed side-by-side and enclosed in a plexiglass chamber. The reference plate was set to 33 °C, whereas the test plate was adjusted to the specific temperature of 20 °C, 28 °C, 38 °C or 46 °C. Animals were gently placed at the center of the apparatus and could move freely between plates. Movements were recorded for 600 s by an infrared camera mounted directly above the enclosed space. The percentage of the time spent on the test plate was recorded with an automated video tracking system.

#### Formalin test

For induction of short-term inflammation, 10 µl of 5% formalin solution was injected subcutaneously into the left hind paw (intraplantar). The spontaneous nocifensive responses (licking, flicking, lifting the injected paw) were recorded for 50 min post injection.

#### CFA injections

Mice were anaesthetized by exposure to isoflurane (1–3%), administered by a calibrated vaporizer. For induction of long-term inflammation, 10 µl of complete Freund’s adjuvant (CFA; Sigma, containing 1 mg/ml of Mycobacteriom tuberculosis) were injected into the right hind paw using a 10 µl-Hamilton syringe (Hamilton company, Reno, Nevada).

### Immunohistochemistry

Mice were anesthetized with intraperitoneal ketamine/xylazine injection (100/10 mg/kg) and transcardially perfused with 0.01 M PBS (pH 7.4) followed by ice-cold 4% paraformaldehyde in 0.14 M Sorensen’s phosphate buffer (pH 7.4). Tissues (sciatic nerve, L3, L4 and L5 DRG and spinal cord) were removed, immersion-fixed in 4% paraformaldehyde (total fixation time 20 min) and cryo-protected with 30% (w/v) sucrose in PBS overnight at 4 °C. Tissue sections were cut on a cryostat at 6 µm (sciatic nerve), 10 µm (DRG), or 20 µm (Spinal Cord) and mounted on slides (Fisher Scientific, Pittsburgh, PA). Sections were immediately processed for detection of target protein or stored at −20 °C for future use.

Sections were incubated in the following solutions: (1) blocking solution (PBS containing 4% normal donkey serum, 2% BSA, 0.1% Triton X-100, and 0.02% sodium azide) for 1 h at room temperature; (2) primary antibodies guinea pig anti-contactin-associated protein (caspr)^66^ (1:2000); rabbit anti-Na_V_1.6 (1:250, Millipore); rabbit anti-panNa_V_ (1:250, Millipore); chicken anti-GFP (1:500, Abcam); mouse anti-NeuN Alex Fluor 488 conjugated (1:500, Millipore); in blocking solution at 4 °C overnight; (3) PBS, 3 × 10 min each; (4) secondary antibodies from Jackson Immuno Research Lab at 1:500 dilution in blocking solution for 1 h at room temperature; (5) PBS, 3 × 10 min. Tissue sections were examined with a Nikon Eclipse E800 fluorescence microscope or a Nikon C1 confocal microscope (Nikon USA, Melville, NY). The specificity of Na_V_1.6 staining described in this study was validated in Scn8a^*medtg*^ mice (global Na_V_1.6 KO) (Supplementary Fig. 2).

### Isolation of DRG neurons

DRG neurons were isolated using protocols described by Dib-Hajj *et al*. 2009 and Huang *et al*. 2013^67,68^. Briefly, DRG tissue was first incubated at 37 °C for 20 min in complete saline solution (CSS) [in mM: 137 NaCl, 5.3 KCl, 1 MgCl2, 25 sorbitol, 3 CaCl2, and 10 Hepes, pH 7.2 adjusted with NaOH] supplemented with 0.5 U/ml Liberase TM (Roche) and 0.6 mM EDTA. The tissue was then incubated for 15 min at 37 °C in CSS containing 0.5 U/ml Liberase TL (Roche), 0.6 mM EDTA, and 30 U/ml papain (Worthington Biochemical). DRGs were centrifuged and triturated in 0.5 ml of DRG culture medium containing 1.5 mg/ml BSA (low endotoxin) and 1.5 mg/ml trypsin inhibitor (Sigma). The cell suspension was filtered through 70 μm nylon mesh cell strainer (Becton Dickinson) to remove debris, and the mesh was then washed twice with 2 ml of DRG culture medium. DRG neurons were pelleted by centrifugation and resuspended in 1 ml of DRG culture medium. Aliquots of 100 µl cell suspension were seeded onto poly-D-lysine/laminin-coated coverslips (BD), and incubated at 37 °C in a 95% air/5% CO_2_ (vol/vol) incubator. After 45 min of incubation, 1.4 ml of DRG culture medium was added into each well. For current-clamp recording, nerve growth factor (50 ng/ml) and glial cell line-derived neurotrophic factor (50 ng/ml) were added to DRG culture medium.

### Whole-cell electrophysiology

The protocols for recording action potential were described previously^68^. Briefly, voltage-clamp recordings were conducted at room temperature (22 ± 1 °C) within 24 hrs after DRG neuron isolation using an EPC-10 amplifier and Patchmaster program (v 53; HEKA Elektronik, Lambrecht/ Pfalz,Germany). Electrodes were fabricated from 1.6 mm outer diameter borosilicate glass micropipettes (World Precision Instruments, Sarasota, FL) and fire-polished using a Sutter Instruments P-97 puller (Novato, CA). Electrodes had a resistance of 0.5–1.2 MΩ. Pipette potential was adjusted to zero before seal formation, and liquid junction potential was not corrected. We implemented 80–90% series resistance compensation to reduce voltage-errors. Linear leak currents were subtracted using the P/N method^69^. We allowed 5 min equilibration period once whole-cell configuration was achieved before we started recording sodium currents. We acquired data at 50 kHz, and filtered it with a low-pass Bessel setting of 2.9 kHz.

We first measured the TTX-S sodium currents in Na_V_1.8-Cre driven Na_V_1.6 knockout (Na_V_1.8^+/Cre^, Na_V_1.6^flox/flox^) and heterozygous (Na_V_1.8^+/Cre^, Na_V_1.6^flox/+^) mice using whole-cell voltage-clamp technique. The pipette solution for voltage-clamp recordings in small neurons contained (in mM): 140 CsF, 10 NaCl, 1 EGTA, 10 dextrose and 10 HEPES, pH 7.3 with CsOH (308 mOsmol/L adjusted with sucrose). The extracellular bath solution for voltage-clamp recording contained (in mM): 20 NaCl, 120 choline chloride, 3 KCl, 1 MgCl_2_, 1 CaCl_2_, 10 HEPES, 5 CsCl, 20 Tetraethylammonium chloride (TEA·Cl), pH 7.32 with NaOH (327 mOsmol/ L). The bath solution contained 0.1 mM CdCl_2_ to block calcium currents and 1 mM 4-Aminopyridine to block potassium currents. Small DRG neurons were held at −100 mV. Total voltage-gated sodium current was measured with 100 ms step depolarizations (−80 to +30 mV) in 5 mV increments (5 s intervals) from the holding potential. The pipette solution for voltage-clamp recordings in large neurons contained (in mM): 140 CsCl, 10 NaCl, 0.5 EGTA, 3 Mg-ATP, and 5 Hepes, pH 7.31 with CsOH (310 mOsmol/L adjusted with dextrose). DRG neuronal membrane is usually more stable with CsF-based pipette solutions which is why CsF is commonly used for routine recordings and for long recording protocols. However, sodium currents are much bigger in large diameter than small diameter neurons and the use of CsCl permits a better clamping of large currents. The extracellular bath solution contained (in mM): 140 NaCl, 3 KCl, 1 MgCl_2_, 1 CaCl_2_, 10 Hepes, 5 CsCl, 20 tetraethylammonium chloride (TEA·Cl), 0.1 CdCl_2_, 5 4-aminopyridine, pH 7.30 with HCl (320 mOsmol/L adjusted with dextrose). Large DRG neurons were held at −70 mV. Current-voltage (I-V) relationship was determined using 100-ms step depolarizations (−65 to +40 mV) in 5 mV increments (5 s intervals) from a 500 ms prepulsing potential at −100 mV.

We performed voltage-clamp studies in large neurons from global Na_V_1.6 null mice (*SCN8a*^medtg^) and their wild-type littermates. The pipette solution contained (in mM): 140 CsF, 10 NaCl, 1 EGTA, 10 dextrose and 10 HEPES, pH 7.3 with CsOH (308 mOsmol/L adjusted with sucrose). Because we recorded resurgent current in these neurons, we decided to use CsF instead for the benefit of maintaining stable cell membrane during lengthy recordings. The bath solution contained (in mM): 30 NaCl, 110 choline chloride, 3 KCl, 1 MgCl_2_, 1 CaCl_2_, 10 HEPES, 5 CsCl, 20 Tetraethylammonium chloride (TEA·Cl), pH 7.33 with NaOH (339 mOsmol/l). Cells were held at −100 mV followed by a series of 100-ms depolarizations from −70 to +10 mV in 5 mV increments. To determine resurgent sodium currents, we used a strong depolarization (20 ms at +10 mV) followed by 200 ms intermediate repolarization pulses to voltages ranging from −10 to −80 mV in 5 mV increments.

TTX (1 µM) was added to the bath solution to isolate the TTX-R currents in both small and large neurons. TTX-S sodium currents in each neuron were obtained by subtracting the TTX-R currents from the total sodium current. Current density was determined by normalizing peak transient currents (*I*_*Trans*_) with cell capacitance.

The protocols for current-clamp recordings were previously described^68^. Briefly, electrodes had a resistance of 0.7–1.5 MΩ. The pipette solution contained (in mM): 140 KCl, 0.5 EGTA, 5 Hepes, and 3 Mg-ATP (pH 7.30 adjusted with KOH and 310 mOsmol/L adjusted with dextrose). The extracellular solution contained (in mM): 140 NaCl, 3 KCl, 2 MgCl_2_, 2 CaCl_2_, 10 Hepes (pH 7.30 adjusted with NaOH and 320 mOsmol/L adjusted with dextrose). We established the whole-cell configuration in the voltage-clamp mode before switching to the current-clamp mode. We included in our analysis cells with stable RMP (<10% variation) negative than −35 mV and overshooting action potentials (>85 mV RMP-to-peak). Input resistance was determined by the slope of a linear fit to hyperpolarizing responses to current steps from −5 to −40 pA in 5 pA increments. Threshold was determined at the first action potential in response to depolarizing current injections (200 ms) in 5 pA increments. Firing frequency was determined by the number of action potentials elicited in response to depolarizing stimuli (500 ms) from 25 to 500 pA in 25 pA increments for small DRG neurons, and from 100 to 2000 pA in 100 pA increments for large DRG neurons. Endogenous Na_V_1.8 currents were assessed by holding neurons at −50 mV^70^. DRG neurons that expressed Na_V_1.8 currents <1 nA and did not generate all-or-none action potentials in response to 200 ms current stimulus were excluded from analysis^71^.

### Statistical analysis

For behavioral studies, we used two-way repeated analysis of variance (ANOVA) followed by Bonferroni post hoc test to assess differences in mechanical and thermal sensitivity between two treatment groups. For immunohistochemical studies, unpaired t-test was used to determine the significance of changes (p < 0.05). For electrophysiological data analysis, we used Fitmaster (HEKA Elektronik) and Origin (Microcal Software, Northampton, MA). Unless otherwise noted, statistical significance was determined (p < 0.05) using an independent t-test. Mann-Whitney test was used in comparison of firing frequencies in response to external current injections. Results are presented as mean ± Standard error of means (SEM).

## Electronic supplementary material

Supplementary Information
