# Supplementary material for: Conditional knockout of NaV1.6 in adult mice ameliorates neuropathic pain
Source: Sci Rep. 2018 Mar 1;8:3845. doi: 10.1038/s41598-018-22216-w (PMC5832877; doi:10.1038/s41598-018-22216-w)
Supplement: Supplementary file 1 — Supplementary Information [file 41598_2018_22216_MOESM1_ESM.docx]

**Conditional knockout of Na_V_1.6 in adult mice ameliorates neuropathic pain**

Lubin Chen^1,2,3^, Jianying Huang^1,2,3^, Peng Zhao^1,2,3^, Anna-Karin Persson^1,2,3^, Fadia B. Dib-Hajj^1,2,3^, Xiaoyang Cheng^1,2,3^, Andrew Tan^1,2,3^, Stephen G. Waxman^1,2,3^ and Sulayman D. Dib-Hajj^1,2,3,*^

^1^Department of Neurology; ^2^Center for Neuroscience & Regeneration Research, Yale University School of Medicine, New Haven, CT 06510; ^3^Rehabilitation Research Center, VA Connecticut Healthcare System, West Haven, CT 06516

**Supplementary Information**

**Methods:**

**Quantitative real-time RT-PCR**

L4 and L5 DRGs from adult wild-type C57Bl/6 (n=3), mice (P12-14) with global knockout of Na_V_1.6 (MED: Scn8a^medtg^; n=4), Na_V_1.8-driven Na_V_1.6 KO mice (Na_V_1.6^Nav1.8^ KO: Na_V_1.6^flox/flox^/ Na_V_1.8^+/Cre^, n= 4) and heterozygous controls (Na_V_1.6^+/flox^/ Na_V_1.8^+/Cre^, n= 4), were freshly dissected and immediately processed for RNA extraction using RNasy Microkit (Qiagen, USA). 300 ng of total RNA from each mouse (pooled L4/5 DRG) was used to generate 1^st^ strand cDNA using Superscript III (Invitrogen, USA). Real time Taqman PCR assays for mouse Scn8a (assay id: Mm00488123_m1) and mouse GAPDH (assay id: Mm99999915_g1) were purchased from Applied Biosystems (USA) and used with Universal Taqman PCR master mix (20X) (Applied Biosystems, USA). One microliter of the cDNA was used as a template in a 20µl reaction and was run in triplicates for each sample. BioRad CFX96 real-time PCR instrument was used and data analysis was conducted using the BioRad CFX Manager software. Normalization and relative expression analysis of Scn8a mRNA in MED samples, with one WT sample as a control, were done using the 2^-ΔΔCt^ method with GAPDH as the internal control. Normalization and relative expression analysis of Scn8a mRNA in Na_V_1.6^Nav1.8^ KO samples, with one heterozygous sample as a control, were done using the 2^-ΔΔCt^ method with GAPDH as the internal control.

**Immunohistochemistry**

Mice were anesthetized with intraperitoneal ketamine/xylazine injection (100/10 mg/kg) and transcardially perfused with 0.01M PBS (pH 7.4) followed by ice-cold 4% paraformaldehyde in 0.14 M Sorensen’s phosphate buffer (pH 7.4). Tissues (sciatic nerve, L3, L4 and L5 DRG and spinal cord) were removed, immersion-fixed in 4% paraformaldehyde (total fixation time 20 min) and cryo-protected with 30% (w/v) sucrose in PBS overnight at 4°C. Tissue sections were cut on a cryostat at 6 µm (sciatic nerve), 10 µm (DRG), or 20 µm (Spinal Cord) and mounted on slides (Fisher Scientific, Pittsburgh, PA). Sections were immediately processed for detection of target protein or stored at -20 °C for future use.

Sections were incubated in the following solutions: (1) blocking solution (PBS containing 4% normal donkey serum, 2% BSA, 0.1% Triton X-100, and 0.02% sodium azide) for 1 h at room temperature; (2) primary antibodies guinea pig anti-contactin-associated protein (caspr) ^69^ (1:2000); rabbit anti-Na_V_1.6 (1:250, Millipore); mouse anti-NeuN Alex Fluor 488 conjugated (1:500, Millipore); rabbit anti-K_V_1.1 (1:250, Alomone); rabbit anti-K_V_1.2 (1:250, Alomone) in blocking solution at 4°C overnight; (3) PBS, 3 × 10 min each; (4) secondary antibodies from Jackson Immuno Research Lab at 1:500 dilution in blocking solution for 1 h at room temperature; (5) PBS, 3 × 10 min. Tissue sections were examined with a Nikon Eclipse E800 fluorescence microscope or a Nikon C1 confocal microscope (Nikon USA, Melville, NY).

**Figures**

**
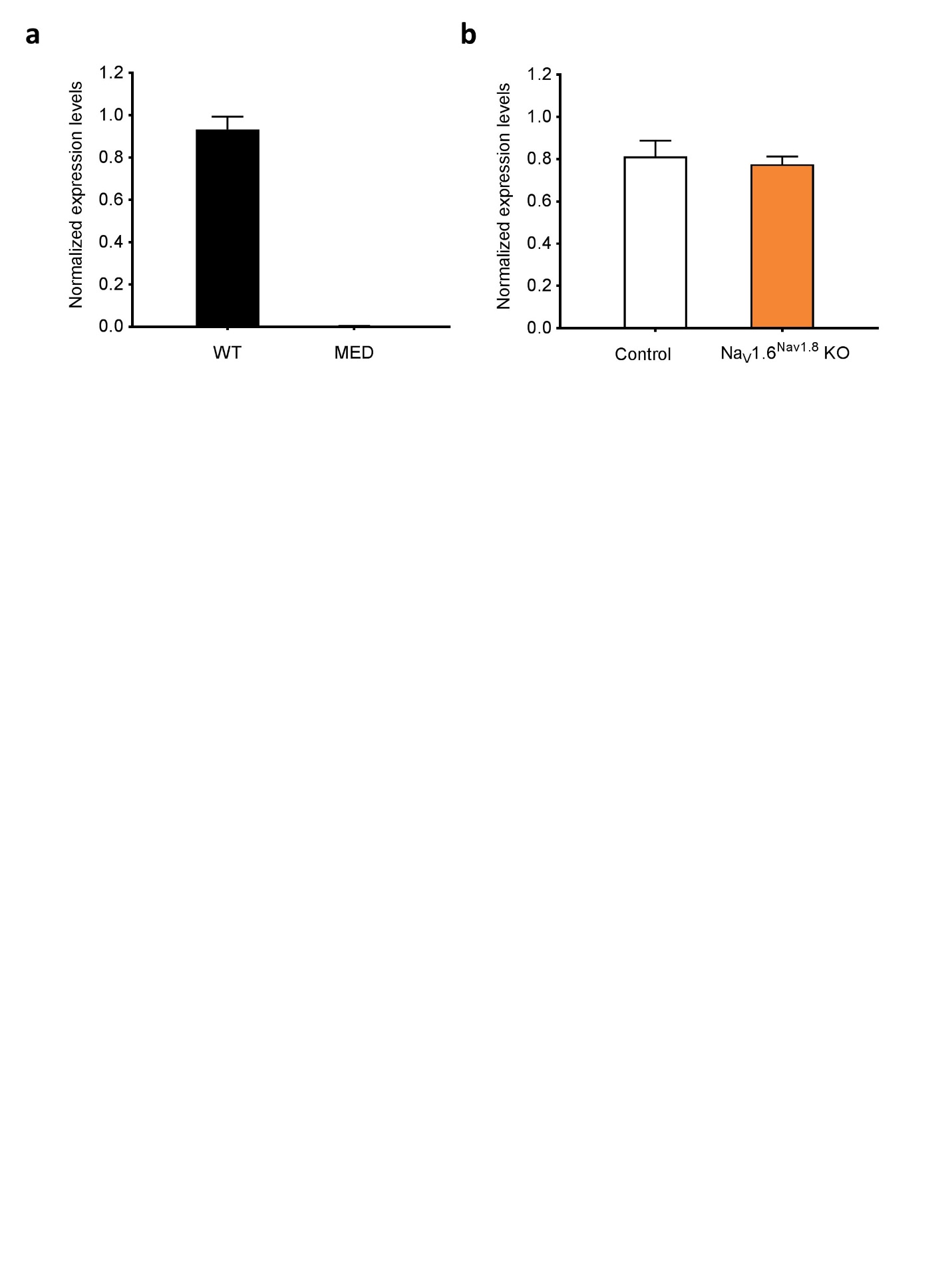
**

**Supplementary Figure 1**: Expression levels of Na_V_1.6 in DRG neurons using quantitative RT-PCR. (**a**) Na_V_1.6 RNA levels detected in DRG from the WT and global knockout mouse strain Scn8a^medtg^ (MED). The normalized gene expression levels of the Scn8a^medtg^ samples represented 0.34% of the WT levels. (**b**) Na_V_1.6 RNA levels in DRG from Na_V_1.6^Nav1.8^ KO mice (Na_V_1.6^flox/flox^/ Na_V_1.8^+/Cre^, n= 4) and heterozygous controls (Na_V_1.6^+/flox^/ Na_V_1.8^+/Cre^, n= 4). No significant difference was observed. We attribute this to high levels of Na_V_1.6 mRNA in Na_V_1.8-negative neurons (primarily large diameter neurons), which dilutes the results.

**
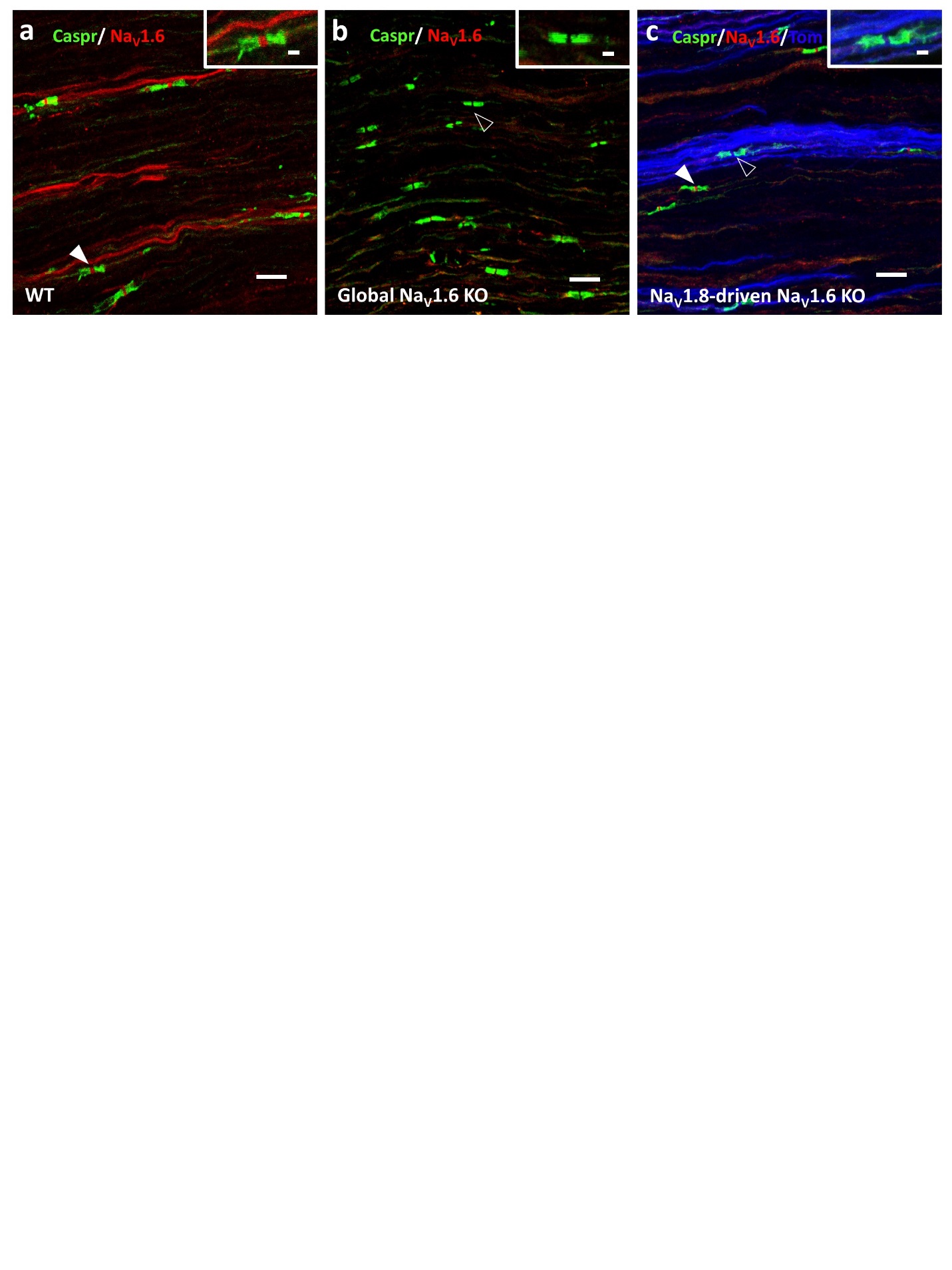
**

**Supplementary Figure 2. Specificity of Na_V_1.6 antibody.** Confocal images of Na_V_1.6 (red) and caspr (green) immune-staining together with tdTomato signal (blue) in sciatic nerve from (**a**) WT, (**b**) global KO, or (**c**) Na_V_1.8-driven Na_V_1.6 KO mice. (**a**) Na_V_1.6 immunostaining was observed along unmyelinated fibers and at nodes of Ranvier (filled arrow head). (**b**) Na_V_1.6 immunostaining was absent at nodes of Ranvier (unfilled arrow head) in global KO mice. (**c**) Na_V_1.6 immunostaining was present at nodes of Ranvier (filled arrow head) along Na_V_1.8-negative A fibers but was absent at nodes of Ranvier (unfilled arrow head) along Na_V_1.8-positive A fibers (blue). Scale bar = 10 µm; Inset scale bar = 2 µm.


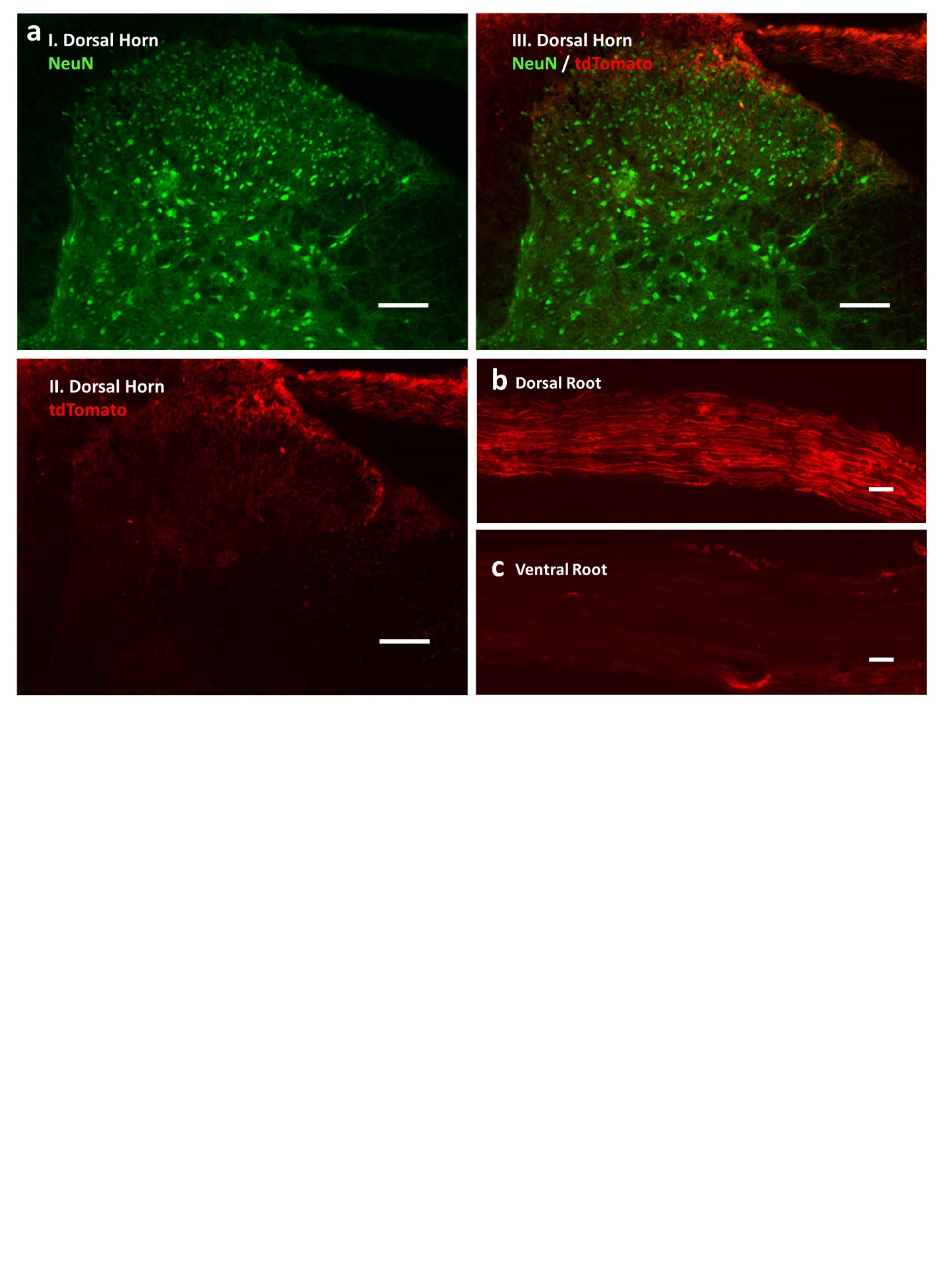


**Supplementary Figure 3. Intrathecally injected AAV does not infect dorsal horn neurons.** Representative images showing tdTomato signal (red) and NeuN immunostaining (green) in (**a**) dorsal horn, (**b**) dorsal root, and (**c**) ventral root. (**aI**) Dorsal horn neurons were marked by NeuN. (**aII**) tdTomato signal was observed in central terminals of primary afferents. **(aIII)** None of the dorsal horn neurons were infected by AAV. (**b**) Presence of strong tdTomato signal along dorsal roots. (**c**) Absence of tdTomato signal along ventral roots. Scale bar = 50 µm.


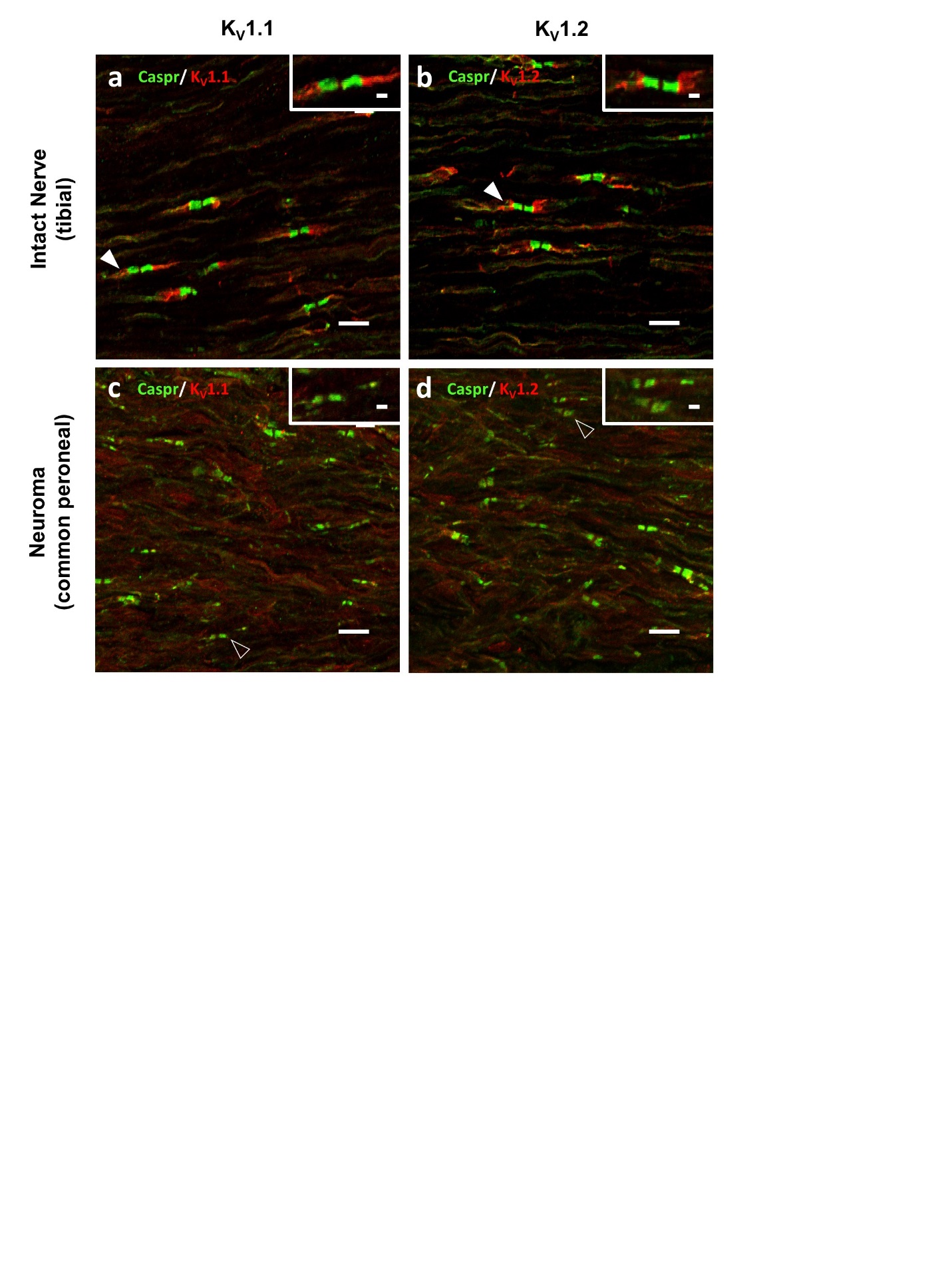


**Supplementary Figure 4.** **Down-regulation of K_V_1.1 and K_V_1.2 in neuroma.** Confocal images of K_V_1.1 and K_V_1.2 (red) with caspr (green) immune-staining in intact nerve (**a-b**) or neuroma (**c-d**). (**a**) K_V_1.1 and (**b**) K_V_1.2 are present at paranodes (filled arrow heads) in intact nerve. (**c**) K_V_1.1 and (**d**) K_V_1.2 are absent at paranodes (unfilled arrow heads) in neuroma. Scale bar = 10 µm; Inset scale bar = 2 µm.

Supplementary Table 1. Antibody List:

| Antibody | Supplier | Catalog No. | Lot No. | Dilution |
| --- | --- | --- | --- | --- |
| rabbit anti-Na_V_1.6 | Millipore | AB5580 | 2689076 | 1:250 |
| rabbit anti-panNa_V_ | Millipore | AB5210 | 2736725 | 1:250 |
| mouse anti-NeuN 488 | Millipore | MAB377X | 2606902 | 1:500 |
| chicken anti-GFP | Abcam | ab13970 | GR89472-23 | 1:500 |
| guinae pig anti-caspr | n/a | n/a | n/a | 1:1000 |
| rabbit anti-K_V_1.1 | Alomone | APC-009 | AG-05 | 1:250 |
| rabbit anti-K_V_1.2 | Alomone | APC-010 | AN-06 | 1:250 |
